# Neuroprotective Effects of Two N‐Benzylamides in Models of Oxidative Stress, Neurite Outgrowth and Neurodegeneration

**DOI:** 10.1155/omcl/3092494

**Published:** 2026-09-17

**Authors:** Subaraja Mamangam, Anantha Krishnan Dhanabalan, Kanika Verma, Mani Iyer Prasanth, Sirikalaya Brimson, James Michael Brimson

**Affiliations:** ^1^ Research, Innovation and International Affairs, Faculty of Allied Health Sciences, Chulalongkorn University, Bangkok 10330, Thailand, chula.ac.th; ^2^ RUBi - Research Unit in Bioinformatics, Department of Biochemistry, Microbiology and Bioinformatics, Rhodes University, Makhanda 6139, South Africa, ru.ac.za; ^3^ Centre for Bioinformatics, Department of Biotechnology, Karpagam Academy ofHigher Education, Coimbatore 641021, India, kahedu.edu.in; ^4^ Department of Molecular Epidemiology, National Institute of Malaria Research, Delhi, India, nimr.org.in; ^5^ Center of Excellence on Natural Products for Neuroprotection and Anti-ageing (Neur-Age Nat Chula), Chulalongkorn University, Bangkok 10330, Thailand, chula.ac.th; ^6^ Department of Molecular Genetics and Cancer, Nitte University Centre for Science Education and Research, Nitte (Deemed to be University), Paneer Campus, Deralakatte, Mangalore 575018, India, nitte.edu.in; ^7^ Department of Pharmacology, KS Hegde Medical Academy, Nitte (Deemed to be University), Deralakatte, Mangalore 575018, India, nitte.edu.in; ^8^ Department of Clinical Microscopy, Faculty of Allied Health Sciences, Chulalongkorn University, Bangkok 10330, Thailand, chula.ac.th

**Keywords:** N-benzylamides, neurite outgrowth, neuroprotection, oxidative stress, sigma-1 receptor

## Abstract

Oxidative stress is a central pathological mechanism in neurodegenerative diseases (NDDs), contributing to mitochondrial dysfunction, impaired proteostasis, and progressive neuronal loss. The sigma‐1 receptor (S1R) is an endoplasmic reticulum (ER)‐resident chaperone that coordinates cellular stress responses and has emerged as a promising therapeutic target for neuroprotection. In this study, we investigated the neuroprotective properties and mechanisms of the structurally related N‐benzylamides, N‐benzylcinnamide (NBCA) and N‐benzylbenzamide (NBBA) using complementary in vitro, in vivo and in silico approaches. Neuroprotective activity was initially evaluated using glutamate‐induced oxidative injury in HT22 hippocampal neurones and H_2_O_2_‐induced oxidative stress in SH‐SY5Y neuroblastoma cells. Both compounds significantly attenuated oxidative injury, although NBCA consistently showed greater efficacy and was therefore selected for detailed mechanistic investigation. NBCA reduced intracellular reactive oxygen species (ROS) production and lipid peroxidation while restoring endogenous antioxidant enzyme activities, mitochondrial membrane potential, ATP production, and cholinergic homeostasis. Immunofluorescence analysis showed that NBCA modulated nuclear factor erythroid 2‐related factor 2 (NRF2) and S1R immunoreactivity after oxidative stress. NBCA also promoted neurite outgrowth in wild‐type (WT) Neuro‐2a cells, whereas these effects were absent in S1R knockout cells, supporting the involvement of S1R‐associated signalling. Molecular docking predicted favourable interactions between NBCA and the stress‐response proteins S1R, BiP and TMEM97, suggesting modulation of interconnected cellular stress‐response networks. In *Caenorhabditis elegans*, NBCA improved resistance to oxidative stress, preserved learning and memory, delayed amyloid‐β‐induced paralysis, and reduced amyloid deposition in transgenic Alzheimer’s disease (AD) models. Collectively, these findings demonstrate that NBCA exerts neuroprotective effects across multiple experimental models by preserving redox homeostasis, mitochondrial function, and neuronal integrity. The convergence of pharmacological, genetic, imaging, computational, and whole‐organism evidence supports the involvement of S1R‐associated signalling in NBCA’s biological activity. Together, these findings demonstrate that modulation of S1R‐associated cellular stress‐response networks is a promising strategy for preserving neuronal function during oxidative stress and neurodegeneration and identify NBCA as a valuable lead compound for future mechanistic and therapeutic investigation.

## 1. Introduction

Neurodegenerative diseases (NDDs) are characterised by the progressive loss of neuronal structure and function, ultimately leading to irreversible neurological impairment. Although disorders such as Alzheimer’s disease (AD), Parkinson’s disease (PD), Huntington’s disease (HD), amyotrophic lateral sclerosis (ALS) and dementia with Lewy bodies (DLB) differ in their clinical manifestations, they share several pathological mechanisms, including oxidative stress, mitochondrial dysfunction, impaired proteostasis, neuroinflammation and progressive neuronal loss [1–3]. Oxidative stress is considered a central driver of neurodegeneration, promoting lipid peroxidation, mitochondrial dysfunction, impaired proteostasis and activation of apoptotic signalling pathways that compromise neuronal survival. Despite advances in symptomatic treatment, effective disease‐modifying therapies remain limited, underscoring the need to identify novel molecular targets that preserve neuronal integrity and function [4].

AD is the most common cause of dementia and is characterised by extracellular amyloid‐β deposition, intracellular accumulation of hyperphosphorylated tau, synaptic dysfunction and progressive neuronal loss [5]. Oxidative stress and mitochondrial impairment contribute to these pathological processes and are closely associated with the disruption of cholinergic neurotransmission, including dysregulation of acetylcholinesterase (AChE) and butyrylcholinesterase (BuChE) activity [6]. Degeneration of basal forebrain cholinergic neurones contributes to the progressive decline in memory, learning and executive function that characterises AD [7]. Although AChE inhibitors provide symptomatic benefit and recently introduced anti‐amyloid therapies can modestly slow cognitive decline in selected patients, available treatments do not fully prevent progressive neuronal dysfunction [8]. There therefore remains a need for therapeutic approaches that simultaneously preserve redox balance, mitochondrial function, proteostasis and cholinergic homeostasis.

The sigma‐1 receptor (S1R) has emerged as a promising therapeutic target for NDDs because of its central role in coordinating cellular stress responses. S1R is an endoplasmic reticulum (ER)‐resident, ligand‐operated chaperone enriched at the mitochondria‐associated membrane (MAM), where it regulates calcium homeostasis, mitochondrial function, proteostasis and cellular adaptation to oxidative stress [9]. Under cellular stress, S1R modulates multiple cytoprotective signalling pathways, including nuclear factor erythroid 2‐related factor 2 (NRF2)‐mediated antioxidant responses, thereby promoting neuronal survival and preserving neuronal function [10]. Consequently, pharmacological modulation of the S1R has attracted considerable interest as a disease‐modifying strategy for neurodegenerative disorders. This concept is supported by the clinical development of S1R agonists such as blarcamesine (Anavex2‐73) for AD [11] and pridopidine for HD [12], which have demonstrated encouraging effects on neuronal function and disease progression [13–16].

N‐benzylamides constitute a diverse class of bioactive compounds with reported central nervous system activity. Among these, N‐benzylcinnamide (NBCA), a natural product isolated from the *Piper submultinerve*, has previously demonstrated neuroprotective activity against amyloid‐β‐induced neuronal injury [17, 18]. However, the molecular mechanisms underlying these neuroprotective effects remain poorly understood. The structurally related analogue N‐benzylbenzamide (NBBA) was therefore included to determine whether the neuroprotective properties observed for NBCA extend to related N‐benzylamide compounds. Elucidating the molecular mechanisms underlying the actions of these compounds may facilitate the rational development of novel therapeutic strategies for AD and related neurodegenerative disorders.

Therefore, the present study investigated the neuroprotective effects and underlying mechanisms of NBCA and NBBA using complementary in vitro, in vivo and in silico approaches. Neuroprotective activity was initially evaluated using glutamate‐induced toxicity in HT22 hippocampal neurones and H_2_O_2_‐induced oxidative stress in SH‐SY5Y neuroblastoma cells, which represent established models of glutamate‐mediated oxidative injury and direct oxidative stress, respectively, and reflect the differential sensitivity and established use of these cell lines in neuroprotection research. Mechanistic studies subsequently examined oxidative stress, antioxidant defence, mitochondrial function, NRF2 signalling, S1R‐associated responses and cholinesterase activity. The effects of NBCA and NBBA on neuronal differentiation and neurodegeneration were further investigated using neurite outgrowth assays and multiple *Caenorhabditis elegans* models, including oxidative stress, learning and memory and amyloid‐β pathology. Finally, molecular docking analyses were performed to explore potential interactions with S1R and other molecular targets.

We hypothesised that NBCA would protect neurones from oxidative stress by preserving mitochondrial function and redox homeostasis through modulation of S1R‐associated stress‐response pathways and that these effects would translate into improved neuronal function in whole‐organism models of neurodegeneration.

## 2. Materials and Methods

### 2.1. Models and Reagents

The human neuroblastoma cell line SH‐SY5Y and the mouse neuroblastoma cell line Neuro‐2a (N2a) were obtained from the Japanese Collection of Research Bioresources (JCRB, Osaka, Japan). The HT22 cell line was a kind gift from Dr. David Schubert at The Salk Institute in La Jolla, CA, USA. S1R knockout (KO) N2a cells were generated using CRISPR/Cas9 technology [19] and kindly provided by Prof. Tsung‐Ping Su (National Institute on Drug Abuse, NIH/DHHS, Baltimore, MD, USA).

*C. elegans* strains CL2006, CL4176 and wild‐type (WT) N2 (Bristol), together with the bacterial food source *Escherichia coli* OP50, were obtained from the Caenorhabditis Genetics Center (CGC, University of Minnesota, USA). The *C. elegans* TM3443 strain was provided by the National Bioresource Project (Tokyo, Japan). Dulbecco’s Modified Eagle Medium (DMEM), foetal bovine serum (FBS), and antibiotics (penicillin and streptomycin) were purchased from Gibco (USA). All chemicals, including hydrogen peroxide (H_2_O_2_), NBCA, NBBA, PRE084 and haloperidol, were obtained from Sigma–Aldrich (USA).

### 2.2. Cell Culture and Maintenance

The SH‐SY5Y, HT22, and N2a cell lines were cultured in DMEM supplemented with 10% FBS, 100 U/mL penicillin, and streptomycin. Cultures were maintained at 37°C in a humidified incubator with 5% CO_2_ and 95% air. The cells were sub‐cultured before reaching 90% confluence, with the media changed accordingly. Cells were separated by centrifugation at 1000 rpm for 5 min at 4 °C and then detached with trypsin‐EDTA. The cell suspension was washed with 1× phosphate‐buffered saline (PBS; pH 7.4), and the resulting pellet was resuspended in fresh DMEM. Cells were then seeded at 3 × 10^4^ cells/cm^2^ for suspension experiments.

### 2.3. Glutamate Toxicity in HT22 Cells

HT22 hippocampal neuronal cells lack functional ionotropic glutamate receptors and are therefore widely used as a model of glutamate‐induced oxidative stress rather than excitotoxicity. In these cells, high concentrations of glutamate inhibit cystine uptake via the cystine/glutamate antiporter (system Xc−), resulting in glutathione depletion, accumulation of reactive oxygen species (ROS), and oxidative cell death. HT22 cells (1 × 10^4^ cells per well) were seeded into 96‐well plates and allowed to adhere overnight before treatment with glutamate (5 mM) in the presence or absence of NBCA or NBBA (50–500 nM). Cells were incubated for 24 h prior to the determination of cell viability using the MTT assay according to the manufacturer’s instructions. The vehicle control consisted of 0.1% dimethyl sulfoxide (DMSO).

### 2.4. H_2_O_2_ Toxicity in SH‐SY5Y Cells

SH‐SY5Y neuroblastoma cells are widely used to investigate neuronal oxidative stress because they retain many neuronal characteristics while responding reproducibly to ROS. Direct exposure to hydrogen peroxide provides a well‐established model of oxidative injury independent of glutamate transport mechanisms, allowing the evaluation of antioxidant and cytoprotective interventions. SH‐SY5Y cells were seeded at 3 × 10^4^ cells per well in six‐well plates and allowed to adhere overnight. Cells were pretreated with NBCA (100, 200 or 500 nM), NBBA, PRE084 or vehicle (0.1% DMSO) before exposure to H_2_O_2_ (200 µM) for 24 h. Cell viability was subsequently determined using the MTT assay according to the manufacturer’s instructions.

### 2.5. Immunofluorescence Staining and Image Analysis

Following treatment, SH‐SY5Y cells grown on glass coverslips were washed with phosphate‐buffered saline (PBS, pH 7.4) and fixed with 4% paraformaldehyde for 10 min. Cells were subsequently washed with PBS, permeabilised with 0.5% Triton X‐100 for 30 min, and blocked with 5% bovine serum albumin (BSA) for 20 min. Cells were incubated overnight at 4°C with either rabbit anti‐sigma‐1 receptor antibody (Invitrogen, Thermo Fisher Scientific. Waltham, MA, USA. Cat. No. 42‐3300, dilution 1:100) or rabbit anti‐NRF2 antibody (Abcam, Cambridge, UK. Cat. No. [EP1808Y] ab76026 Dilution 1:200). Following washing with PBS, cells were incubated for 1 h with Anti‐rabbit IgG (H + L), F(ab’)_2_ Fragment (Alexa Fluor 488 Conjugate) secondary antibody (Cell Signaling Technology. Danvers, MA, USA, Cat. No. 4412; 1:500). Nuclei were counterstained with DAPI (4^′^,6‐diamidino‐2‐phenylindole) (1 mg/mL); (Cell Signaling Technology. Danvers, MA, USA) for 5 min. The coverslips were mounted and imaged using a Cytation 7 Cell Imaging Multi‐Mode Reader (Agilent BioTek, Europe). All images within an experiment were acquired using identical microscope settings, including objective magnification, exposure time, LED intensity and camera gain, without adjustment between treatment groups.

Fluorescence images were analysed using ImageJ software [20]. Images were converted to 32‐bit grayscale, and background fluorescence was subtracted before analysis. The Alexa Fluor 488 channel was thresholded using the default threshold algorithm with identical threshold settings applied to all images acquired within the same experiment. Immunoreactivity was quantified using ImageJ. For each treatment group, 10 randomly selected, non‐overlapping fields of view were analysed. Alexa Fluor 488 images were converted to 32‐bit grayscale images, and a fixed fluorescence threshold was applied to all images within each experiment. Threshold‐defined cellular particles were identified using the Analyse Particles function, and the mean fluorescence intensity, expressed in arbitrary units (AU), was calculated for each cell. Mean cellular fluorescence values were then averaged for each field of view and used for statistical analysis. Data were presented as mean fluorescence intensity per cell. The specificity of immunostaining was confirmed using samples processed in parallel, omitting either the primary or secondary antibody.

### 2.6. Neuro2A Neurite Outgrowth Assay

S1R‐expressing WT and S1R knockout (S1R‐KO) Neuro‐2a (N2a) cells were seeded at a density of 2000 cells/cm^2^ in six‐well plates and incubated overnight to allow cell attachment. The culture medium was then replaced with low‐serum DMEM (<0.1% FBS) to induce neuronal differentiation. Cells were treated with NBCA or NBBA (50 nM–1 µM) or vehicle (0.1% DMSO) and incubated for 24 h at 37°C in a humidified atmosphere containing 5% CO_2_.

Each treatment condition was evaluated in three independent biological experiments, with three replicate wells per treatment in each experiment. From each well, four randomly selected non‐overlapping fields of view were imaged using phase‐contrast microscopy. Neurite outgrowth was quantified using ImageJ software [20], with neurites defined as cellular extensions at least 1.5 times the cell body diameter [21]. All neurite‐bearing cells within each field of view (typically 50–100 cells per field) were measured. Measurements from the four fields were averaged for each well, and the three replicate wells were subsequently averaged to generate a single value for each biological experiment. Statistical analyses were therefore performed using data from three independent biological experiments (*n* = 3).

### 2.7. Intracellular ROS Measurement

SH‐SY5Y cells were seeded and treated as described above. Following treatment, intracellular ROS production was determined using the fluorescent probe 2^′^,7^′^‐dichlorodihydrofluorescein diacetate (H_2_DCFDA). Cells were incubated with H_2_DCFDA under the manufacturer’s recommended conditions, and fluorescence intensity was measured using a fluorescence microplate reader at the appropriate excitation and emission wavelengths. ROS levels were expressed as relative fluorescence intensity.

### 2.8. Protein Determination

Following treatment, the cells were harvested and lysed in RIPA buffer. Cell lysates were centrifuged at 1000 rpm for 10 min at 4°C, and the supernatants were collected. The total protein concentration was determined using the Bradford assay. Briefly, 100 μL of cell lysate was mixed with 300 μL of Bradford reagent and incubated for 5 min before the absorbance was measured at 595 nm. Protein concentrations were calculated using a BSA standard curve.

### 2.9. Superoxide Dismutase (SOD) Activity

SOD activity was determined from protein lysates using the method of Sun et al. [22] with some modifications. Enzyme activity was normalised to the total protein concentration and expressed as U/mg protein/min.

### 2.10. Catalase (CAT) Activity

CAT activity was determined according to the method described by Aebi [23] with some modifications. Enzyme activity was normalised to the total protein concentration and expressed as U/mg protein/min.

### 2.11. Glutathione Peroxidase (GPx) Activity

GPx activity was determined using the method of Paglia and Valentine [24] with some modifications. Enzyme activity was normalised to the total protein concentration and expressed as U/mg protein/min.

### 2.12. Glutathione Reductase (GR) Activity

GR activity was determined according to Beutler et al. [25] with some modifications. Enzyme activity was normalised to the total protein concentration and expressed as U/mg protein/min.

### 2.13. Lipid Peroxidation

Lipid peroxidation was assessed by measuring thiobarbituric acid‐reactive substances (TBARS) using the method of Ohkawa et al. [26] with some modifications. Lipid peroxidation levels were normalised to the total protein concentration and expressed as mol/mg protein/min.

### 2.14. Caenorhabditis elegans

The CL2006, TM3443, WT N2 and CL4176 strains were routinely cultured at 15°C on a nematode growth medium (NGM). The experimental procedures involved using young adult worms.

### 2.15. Lifespan Assay

Lifespan analysis was conducted as previously described [27] on both WT and mutant strains of *C. elegans*. Age‐synchronised worms (10 per well) were transferred into a 24‐well plate containing M9 buffer, with 25 µL of 5‐fluoro‐2^′^‐deoxyuridine (FUDR) added to each medium. The worms were treated with drugs (at specified concentrations), DMSO was used as the vehicle control, and viability was evaluated by counting the number of live individuals at specific time points.

### 2.16. Chemotaxis and Cognition Behaviours

Naïve chemotaxis, learning, and memory assays were performed as previously described [28, 29]. Briefly, synchronised *C. elegans* strains (N2 [referred to from here on as WT], CL4176, CL2006 and TM3443) were treated with the specified compounds and cultured on NGM plates at 15°C for 36 h. Treated worms were subsequently maintained under standard growth conditions.

For chemotaxis assays, synchronised worms (*n* = 10) were washed with M9 buffer and transferred to the centre of the assay plates. Each plate contained 1 µL of 10% butanone (attractant) and 1 µL of 95% ethanol (control), each spotted with 0.5 µL of 0.5 M sodium azide (paralytic agent) in the opposing quadrants. After a 1‐h of incubation, the number of paralysed worms in each quadrant was recorded. The chemotaxis index (CI) was calculated as follows:

CI=T12+T−C12+CTotal worms scored.

For learning and memory assays, remaining worms were first starved in M9 buffer for 1 h and then conditioned on OP50‐seeded NGM plates exposed to 10% butanone vapour for 1 h. After conditioning, worms were divided into three groups: one was tested immediately (*t* = 0), and two were re‐incubated on OP50‐seeded NGM plates without butanone for 1 and 2 h, respectively (*t* = 1 and *t* = 2). Following each time point, worms were tested as described above, and the CI was recalculated.

Locomotor activity was assessed by carefully touching the worms with a platinum wire. Individuals displaying only head movement or failing to produce a complete body sinusoidal wave were classified as paralysed.

### 2.17. Paralysis Assay

Paralysis assays were performed as previously described [30, 31] with minor modifications. Age‐synchronised CL2006 and CL4176 *C. elegans* were treated in a liquid medium containing the indicated compounds or vehicle control for 36 h before being transferred to fresh NGM plates. The worms were maintained at 15°C throughout the experiment.

Paralysis was assessed every 5 days over a 20‐day observation period. At each time point, only living adult worms were scored; larvae, visibly smaller progeny and dead worms were excluded from the analysis. Worms were considered paralysed if they displayed only head movement or failed to generate a complete sinusoidal body wave following gentle stimulation with a platinum wire. The percentage of paralysed worms was calculated as follows:

% Paralysed worms=100×Number of paralysed adult wormsTotal number of adult worms scored.

### 2.18. Thioflavin Staining

Following the treatment period, CL2006 worms were fixed in 4% paraformaldehyde for 24 h at 4°C and subsequently permeabilised in a solution containing 5% freshly prepared β‐mercaptoethanol, 1% Triton X‐100 and 125 mM Tris at 37°C for 24 h. The animals were then stained with 0.125% Thioflavin S in 50% ethanol for 5–10 min, destained, and mounted on glass slides for imaging. Confocal microscopy was performed using a 40× objective under standardised exposure parameters with a digital camera. The number of Thioflavin S‐positive deposits in the anterior region of each worm was observed.

### 2.19. Statistical Analysis

Data are presented as the mean ± standard error of the mean (SEM). Statistical comparisons between control and treatment groups were performed using one‐way analysis of variance (ANOVA), followed by Tukey’s or Dunnett’s *post hoc* multiple comparison test (as described). A *p*‐value of less than 0.05 was considered statistically significant. All statistical analyses were performed using GraphPad Prism.

## 3. Results

### 3.1. NBCA and NBBA Protect Neuronal Cells Against Glutamate‐ and H_2_O_2_‐Induced Oxidative Injury

To evaluate the neuroprotective potential of NBCA and NBBA, their effects were initially examined using two complementary in vitro models of oxidative neuronal injury. HT22 hippocampal neurones were exposed to glutamate‐induced oxidative stress, an established model in which glutamate depletes intracellular glutathione and promotes ROS‐mediated cell death. To confirm that any protective effects were not restricted to this model, SH‐SY5Y human neuroblastoma cells were subsequently challenged with H_2_O_2_‐induced oxidative stress, which induces direct ROS generation and mitochondrial dysfunction. Cell viability was assessed using the MTT assay.

**Figure 1:**
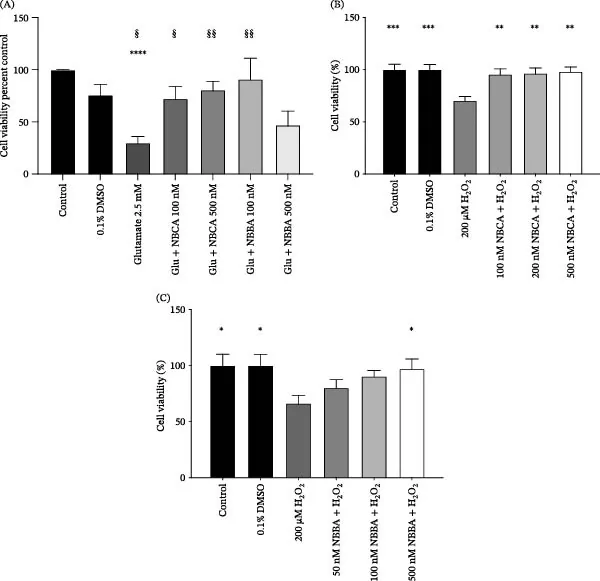
NBCA and NBBA protect neuronal cells against glutamate‐ and H_2_O_2_‐induced oxidative injury. (A) NBCA and NBBA in the glutamate‐induced cytotoxicity assay in HT22 cells. Data represented as mean ± SEM. ANOVA followed by Tukey’s multiple comparisons test  ^∗∗∗∗^ Control vs. Glutamate 2.5 mM *p*  < 0.0001, Control vs. 0.1% DMSO ns, Glutamate 2.5 mM vs. 0.1% DMSO *p*  < 0.05, §§ Glutamate 2.5 mM vs. Glu + NBCA 500 nM *p*  < 0.01, § Glutamate 2.5 mM vs. Glu + NBCA 100 nM ns, Glutamate 2.5 mM vs. Glu + NBBA 500 nM *p*  < 0.05, §§ Glutamate 2.5 mM vs. Glu + NBBA 100 nM *p*  < 0.01. (B) SH‐SY5Y cells exposed to H_2_O_2_, treated with NBCA. ANOVA followed by Tukey’s multiple comparisons test.  ^∗∗^ vs. H_2_O_2_ *p*  < 0.01,  ^∗∗∗^ vs. H_2_O_2_ *p*  < 0.001. (C) SH‐SY5Y cells exposed to H_2_O_2_, treated with NBBA. ANOVA followed by Tukey’s multiple comparisons test.  ^∗^ vs. H_2_O_2_ *p*  < 0.05.

Exposure of HT22 cells to 5 mM glutamate significantly reduced the cell viability compared with untreated control cells (Figure 1A). Co‐treatment with NBCA significantly attenuated glutamate‐induced cytotoxicity at both 100 and 500 nM, restoring cell viability to values close to the control. NBBA likewise significantly protected HT22 cells against glutamate‐induced injury, with 100 nM producing the greatest protective effect, whereas protection was less pronounced at 500 nM.

To determine whether these neuroprotective effects extended to a second model of oxidative injury, SH‐SY5Y cells were exposed to 200 µM H_2_O_2_. H_2_O_2_ treatment significantly reduced cell viability (ANOVA followed by Dunnett’s *post hoc* test for significance, *p*  < 0.001) (Figure 1B). Co‐treatment with NBCA significantly increased cell viability at 100, 200 and 500 nM, restoring viability ANOVA followed by Dunnett’s *post hoc* test for significance, *p*  < 0.01) (Figure 1B). NBBA also significantly attenuated H_2_O_2_‐induced cytotoxicity (Figure 1C), with cell viability increasing in a concentration‐dependent manner, only reaching statistical significance at 500 nM (ANOVA followed by Dunnett’s *post hoc* test for significance *p*  < 0.05).

To exclude the possibility that the observed increases in cell viability reflected direct effects of the compounds on basal cell growth or survival, the effects of NBCA and NBBA alone were evaluated in SH‐SY5Y cells (Supporting Information 1: Figures S1 and S2). Neither compound significantly affected cell viability at concentrations between 100 and 500 nM. However, concentrations of 600 nM and above produced a progressive reduction in cell viability, indicating concentration‐dependent cytotoxicity at higher doses. Consequently, subsequent mechanistic studies were performed using NBCA concentrations between 100 and 500 nM, which showed no detectable cytotoxicity.

### 3.2. NBCA Attenuates Oxidative Stress and Restores Endogenous Antioxidant Defences

Having established that both NBCA and NBBA protected neuronal cells against oxidative injury, subsequent mechanistic studies focused on NBCA to investigate the cellular pathways underlying its neuroprotective activity. NBCA was selected because it demonstrated consistent neuroprotective efficacy across both HT22 and SH‐SY5Y cell models and has previously been reported to protect against amyloid‐β‐induced neuronal injury. As oxidative stress represents a central mechanism underlying H_2_O_2_‐induced neuronal damage, we next examined the effects of NBCA on intracellular ROS production, lipid peroxidation and endogenous antioxidant defence systems in SH‐SY5Y cells.

**Figure 2:**
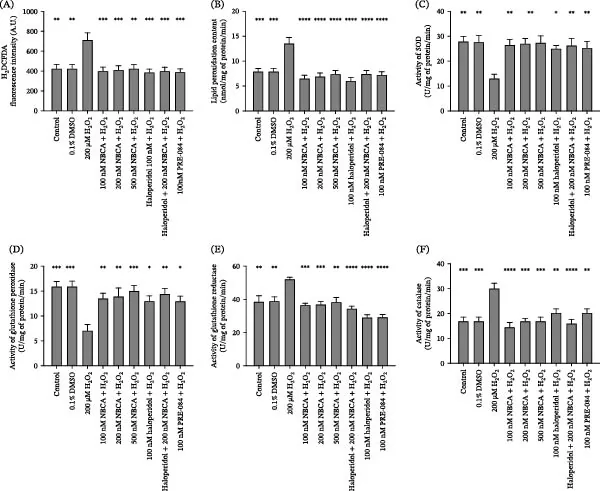
The effects of H_2_O_2_ on ROS related activity in SH‐SY5Y cells. Data represented as mean ± SEM. (A) Reactive oxygen species, measured with H_2_DCFDA fluorescence intensity. (B) Lipid Peroxidation content. (C) Superoxide dismutase activity. (D) Glutathione Peroxidase activity. (E) Glutathione reductase activity. (F) Catalase activity. ANOVA followed by Dunnett’s *post hoc* statistical test for significance versus 200 µM H_2_O_2_ ^∗^ *p*  < 0.05;  ^∗∗^ *p*  < 0.01;  ^∗∗∗^ *p*  < 0.001;  ^∗∗∗∗^ *p*  < 0.0001.

Exposure of SH‐SY5Y cells to 200 µM H_2_O_2_ significantly increased intracellular ROS production compared with vehicle‐treated cells, as determined by H_2_DCFDA fluorescence (Figure 2A). Treatment with NBCA significantly reduced ROS levels at 100, 200 and 500 nM, with the greatest reduction observed at 500 nM. Similarly, H_2_O_2_ significantly increased lipid peroxidation, whereas NBCA treatment significantly reduced it across all concentrations tested (Figure 2B).

H_2_O_2_ exposure also significantly altered endogenous antioxidant enzyme activities (Figure 2C–F). SOD, GPx, GR and CAT activities were all significantly reduced following H_2_O_2_ treatment compared with vehicle‐treated cells. Treatment with NBCA significantly restored the activity of each antioxidant enzyme, with the largest effects generally observed at 500 nM.

### 3.3. NBCA Preserves Mitochondrial Function and Attenuates Cellular Senescence Following H_2_O_2_ Exposure

As oxidative stress is closely associated with mitochondrial dysfunction, the effects of NBCA on mitochondrial membrane potential, ATP production and cellular senescence were subsequently investigated.

**Figure 3:**
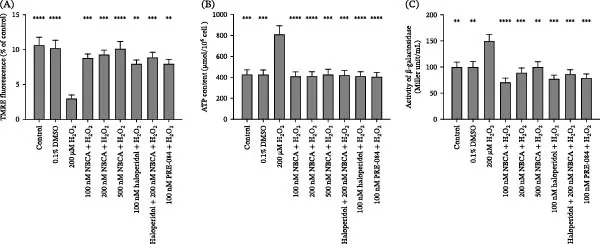
The effects of NBCA on mitochondrial‐related functions in SH‐SY5Y cells treated with H_2_O_2_. Data represented as mean ± SEM. (A) TMRE fluorescence measurement of mitochondrial membrane potential. (B) ATP content of the cells. (C) Beta‐galactosidase activity. ANOVA followed by Dunnett’s *post hoc* statistical test for significance versus 200 µM H_2_O_2_ ^∗^ *p*  < 0.05;  ^∗∗^ *p*  < 0.01;  ^∗∗∗^ *p*  < 0.001;  ^∗∗∗∗^ *p*  < 0.0001 (*n* = 3).

Exposure of SH‐SY5Y cells to H_2_O_2_ significantly reduced the mitochondrial membrane potential, as determined by TMRE fluorescence (Figure 3A). Treatment with NBCA significantly restored mitochondrial membrane potential at all concentrations tested, with the greatest effect observed at 500 nM.

Consistent with the reduction in mitochondrial membrane potential, H_2_O_2_ exposure also significantly decreased the intracellular ATP content compared with vehicle‐treated cells (Figure 3B). NBCA treatment significantly increased ATP levels at 100, 200 and 500 nM, restoring ATP content towards that observed in untreated control cells.

H_2_O_2_ treatment significantly increased β‐galactosidase activity compared with the vehicle‐treated control (Figure 3C). Treatment with NBCA significantly reduced β‐galactosidase activity at all concentrations tested.

### 3.4. NBCA Modulates Cholinesterase Activity Following H_2_O_2_‐Induced Oxidative Stress

As disruption of cholinergic neurotransmission is a characteristic feature of AD and is closely associated with oxidative neuronal injury, the effects of NBCA on the activities of the cholinergic enzymes AChE and BuChE were subsequently investigated in H_2_O_2_‐treated SH‐SY5Y cells.

**Figure 4:**
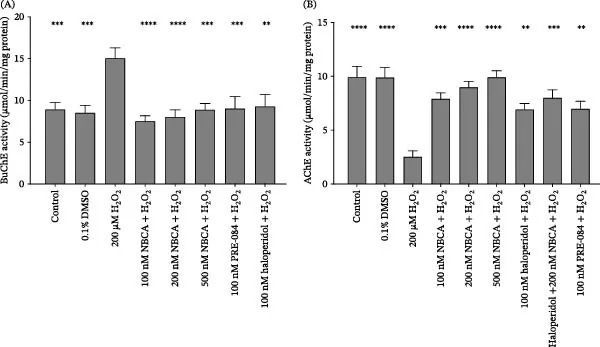
The effect of H_2_O_2_ on (A) AChE and (B) BuChE activity in SH‐SY5Y cells. Data represented as mean ± SEM. ANOVA followed by Dunnett’s *post hoc* statistical test for significance versus 200 µM H_2_O_2_ ^∗^ *p*  < 0.05;  ^∗∗^ *p*  < 0.01;  ^∗∗∗^ *p*  < 0.001;  ^∗∗∗∗^ *p*  < 0.0001 (*n* = 3).

H_2_O_2_ exposure significantly reduced AChE activity compared with vehicle‐treated cells (Figure 4A). Treatment with NBCA significantly restored AChE activity at all concentrations tested, with the greatest recovery observed at 500 nM. Treatment with haloperidol alone also significantly increased AChE activity compared with the H_2_O_2_‐treated group. Co‐treatment with haloperidol and NBCA partially restored AChE activity, although the response was lower than that observed with 200 nM NBCA alone. Similarly, treatment with PRE084 significantly increased AChE activity compared with H_2_O_2_‐treated cells.

H_2_O_2_ treatment significantly increased BuChE activity compared with vehicle‐treated cells (Figure 4B). NBCA significantly attenuated this increase at 100, 200 and 500 nM, restoring BChE activity towards control levels. PRE084 and haloperidol also reduced H_2_O_2_‐induced BChE activity compared with the H_2_O_2_‐treated group.

### 3.5. NBCA Enhances NRF2‐Associated Antioxidant Responses Following Oxidative Stress

As oxidative stress is a major driver of neuronal dysfunction, we next investigated whether the neuroprotective effects of NBCA were associated with the activation of endogenous antioxidant defence pathways. NRF2 is a key regulator of the cellular antioxidant response, controlling the expression of numerous cytoprotective genes involved in maintaining redox homeostasis. Therefore, NRF2 expression was examined by immunofluorescence following H_2_O_2_ exposure and NBCA treatment.

**Figure 5:**
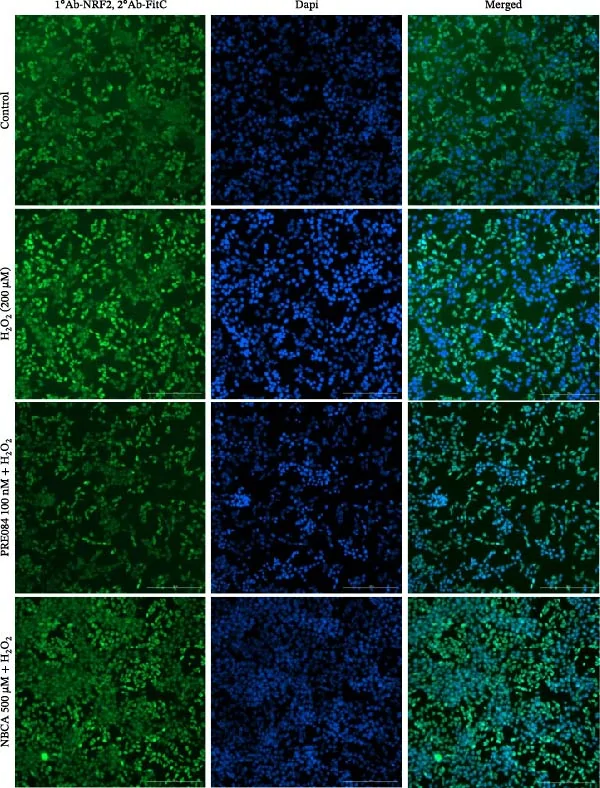
NRF2 specific reactivity in immunofluorescence assay of SH‐SY5Y cells in the presence and absence of 200 µM H_2_O_2_ and or NBCA.

H_2_O_2_ exposure markedly reduced NRF2 immunoreactivity compared with untreated control cells (Figure 5). Treatment with NBCA (500 nM) substantially increased NRF2 fluorescence intensity following H_2_O_2_ exposure, restoring signal intensity towards that observed in untreated controls. Similarly, treatment with the S1R agonist PRE084 increased NRF2 immunoreactivity relative to H_2_O_2_‐treated cells. Representative fluorescence images are shown in Figure 5, with corresponding bright‐field images presented in Supporting Information 1: Figure S3.

Evaluation of additional treatment groups demonstrated a concentration‐dependent increase in NRF2 immunoreactivity following NBCA treatment, with the greatest response observed at 500 nM (Supporting Information 1: Figure S4). In contrast, co‐treatment with haloperidol attenuated the NBCA‐induced increase in NRF2 immunoreactivity, while haloperidol alone produced only a modest increase compared with H_2_O_2_‐treated cells (Supporting Information 1: Figure S4).

**Figure 6:**
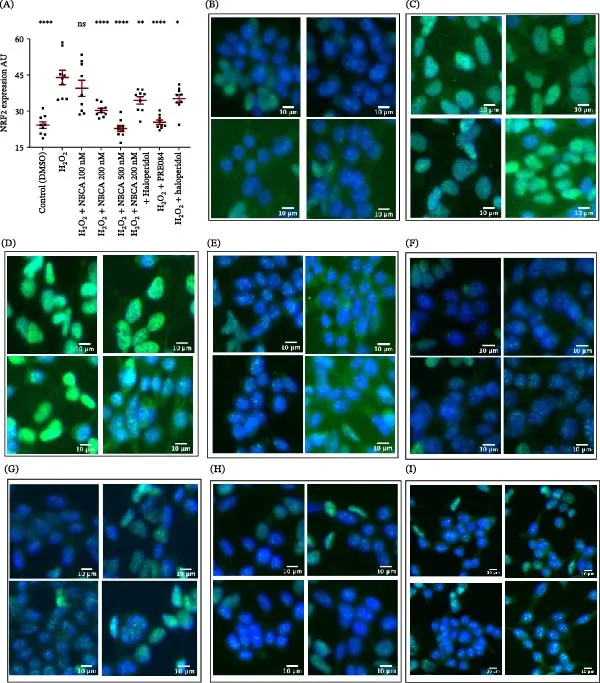
Analysis of immunofluorescence for total NRF2 and increased‐magnification images showing the localisation of NRF2. Data represented as mean ± SEM (*n* = 10). (A) Quantification of total NRF2 fluorescence. (B) Conrtol (DMSO 0.1%) localisation of NRF2. (C) 200 µM H_2_O_2_ localisation of NRF2. (D) 200 µM H_2_O_2_ + 100 nM NBCA localisation of NRF2. (E) 200 µM H_2_O_2_ + 200 nM NBCA localisation of NRF2. (F) H_2_O_2_ + 500 nM NBCA localizaiont of NRF2. (G) 200 µM H_2_O_2_ + 200 nM NBCA + 100 nM. (H) 100 nM Haloperidol + 200 µM H_2_O_2_. (I) 200 µM H_2_O_2_ + 100 nM PRE084. ANOVA followed by Dunnett’s statistical test of significance  ^∗^ *p*  < 0.05;  ^∗∗^ *p*  < 0.01;  ^∗∗∗^ *p*  < 0.001;  ^∗∗∗∗^ *p*  < 0.0001 (*n* = 3).

Representative immunofluorescence images are shown in Figure 5, with corresponding bright‐field images presented in Supporting Information 1: Figure S3. Quantitative analysis of mean NRF2 immunofluorescence intensity per cell, together with higher‐magnification representative images illustrating the staining patterns across all treatment groups, is presented in Figure 6 and Supporting Information 1: Figure S4.

Quantification of NRF2 immunoreactivity, measured as mean Alexa Fluor 488 fluorescence intensity (AU) per cell, confirmed the observations from the representative images (Figure 6A). Fluorescence intensity was quantified using ImageJ by converting images to 32‐bit format, applying a fixed fluorescence threshold, and measuring the mean fluorescence intensity of threshold‐defined cellular particles. H_2_O_2_ treatment significantly increased NRF2 immunoreactivity compared with vehicle‐treated cells. NBCA produced a concentration‐dependent reduction in NRF2 immunoreactivity, with significant decreases observed at 200 and 500 nM. PRE084 similarly reduced NRF2 immunoreactivity compared with the H_2_O_2_‐treated group, whereas co‐treatment with haloperidol attenuated the NBCA effect. Haloperidol alone also significantly reduced NRF2 immunoreactivity compared with H_2_O_2_‐treated cells.

Higher‐magnification images demonstrated distinct differences in the distribution and intensity of NRF2 immunoreactivity between treatment groups (Figure 6B–I). Vehicle‐treated cells exhibited relatively weak, diffuse NRF2 immunoreactivity (Figure 6B), whereas H_2_O_2_ exposure markedly increased cellular NRF2 immunoreactivity (Figure 6C). Treatment with NBCA progressively reduced NRF2 immunoreactivity at 100, 200 and 500 nM (Figure 6D–F), producing staining patterns that more closely resembled those of untreated control cells. Co‐treatment with haloperidol partially attenuated the effect of NBCA (Figure 6G), while haloperidol alone (Figure 6H) and PRE084 (Figure 6I) also altered NRF2 immunoreactivity compared with H_2_O_2_‐treated cells.

**Figure 7:**
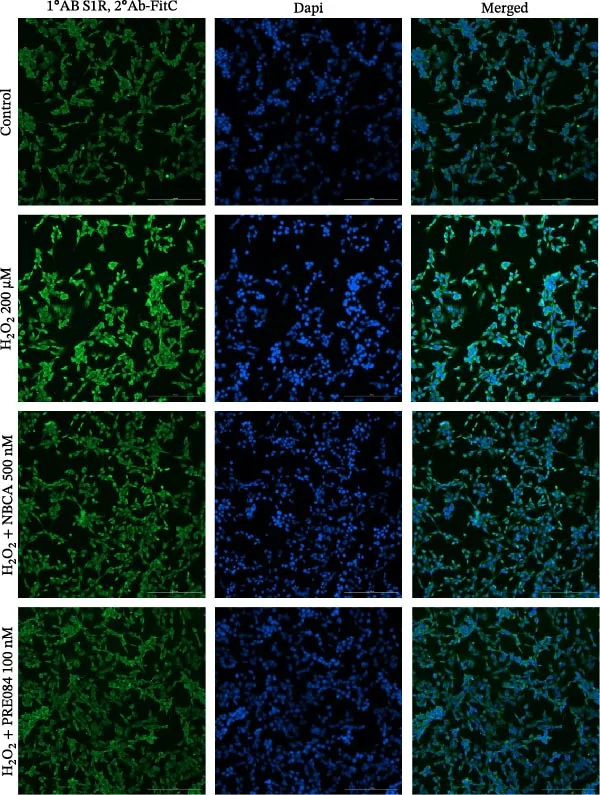
Total S1R reactivity with S1R‐specific primary antibody in immunofluorescence assay of SH‐SY5Y cells treated with H_2_O_2_ and/or NBCA.

To determine whether the effects of NBCA on oxidative stress and NRF2‐associated responses were accompanied by changes in sigma‐1 receptor (S1R) expression, S1R immunoreactivity was examined by immunofluorescence following H_2_O_2_ exposure and pharmacological treatment. Representative immunofluorescence images are shown in Figure 7, with corresponding bright‐field images presented in Supporting Information 1: Figure S5. Additional representative images from the remaining treatment groups are shown in Supporting Information 1: Figure S6. The specificity of S1R immunostaining was confirmed using primary antibody omission controls, which demonstrated negligible Alexa Fluor 488 fluorescence in the absence of the primary antibody (Supporting Information 1: Figure S7).

Representative images demonstrated differences in the intensity and distribution of S1R immunoreactivity between treatment groups. Untreated control cells exhibited relatively weak diffuse S1R immunoreactivity, whereas H_2_O_2_ exposure increased it. Treatment with NBCA (500 nM) reduced S1R immunoreactivity towards control levels. The PRE084 treatment produced a similar staining pattern following H_2_O_2_ exposure. Lower concentrations of NBCA together with the haloperidol‐treated groups are presented in Supporting Information 1: Figure S6.

**Figure 8:**
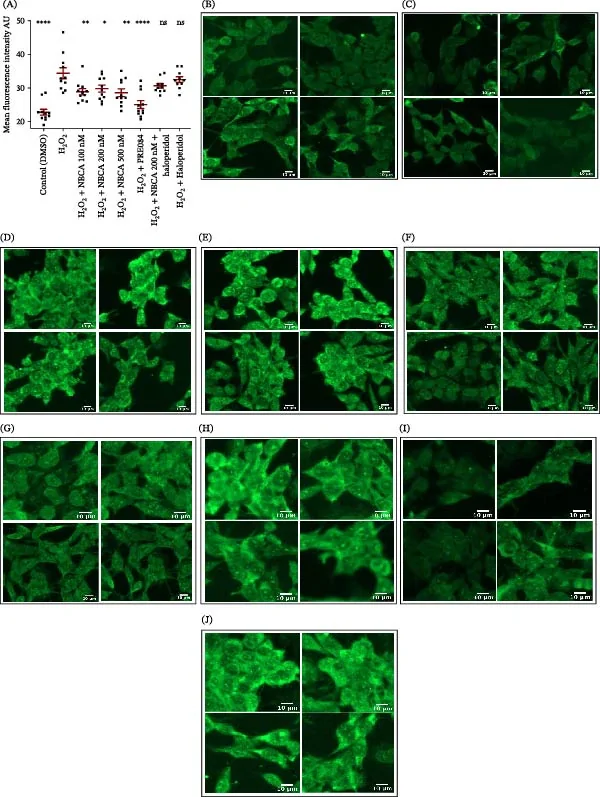
Analysis of immunofluorescence for total S1R and increased magnification images showing the distribution of S1R immunoreactivity. (A) Quantification of total S1R antibody reactivity. Data represented as mean ± SEM. (B) Control. (C) DMSO 0.1%. (D) 200 µM H_2_O_2_. (E) 200 µM H_2_O_2_ + 100 nM NBCA. (F) 200 µM H_2_O_2_ + 200 nM NBCA. (G) 200 µM H_2_O_2_ + 500 nM NBCA. (H) 200 nM NBCA + 200 µM H_2_O_2_ + 100 nM Haloperidol. (I) 200 µM H_2_O_2_ + 100 nM PRE084. (J) 100 nM Haloperidol + 200 µM H_2_O_2_. ANOVA followed by Dunnett’s statistical test of significance  ^∗^ *p*  < 0.05;  ^∗∗^ *p*  < 0.01;  ^∗∗∗^ *p*  < 0.001;  ^∗∗∗∗^ *p*  < 0.0001 (*n* = 3).

Quantification of S1R immunoreactivity, measured as mean Alexa Fluor 488 fluorescence intensity (AU) per cell, confirmed the observations from the representative images (Figure 8A). H_2_O_2_ treatment significantly increased the S1R immunoreactivity compared with vehicle‐treated cells. Treatment with NBCA significantly reduced S1R immunoreactivity at 100, 200 and 500 nM, with the greatest reduction observed at 500 nM. PRE084 similarly reduced S1R immunoreactivity compared with the H_2_O_2_‐treated group. In contrast, co‐treatment with haloperidol partially attenuated the effect of NBCA, whereas haloperidol alone did not significantly alter S1R immunoreactivity compared with H_2_O_2_‐treated cells.

Higher‐magnification images demonstrated differences in the distribution and intensity of S1R immunoreactivity between treatment groups (Figure 8B–J). Vehicle‐treated control and DMSO‐treated cells exhibited relatively weak diffuse S1R immunoreactivity (Figure 8B,C). Exposure to H_2_O_2_ markedly increased the S1R immunoreactivity (Figure 8D). NBCA treatment progressively reduced S1R immunoreactivity at 100, 200 and 500 nM (Figure 8E–G), producing staining patterns that more closely resembled those of the control groups. Co‐treatment with haloperidol partially attenuated the effect of NBCA (Figure 8H), whereas PRE084 similarly reduced S1R immunoreactivity following H_2_O_2_ exposure (Figure 8I). Haloperidol treatment alone did not significantly alter S1R immunoreactivity compared with H_2_O_2_‐treated cells (Figure 8J).

### 3.6. NBCA Promotes Neurite Outgrowth Through an S1R‐Dependent Mechanism

To determine whether the modulation of S1R immunoreactivity by NBCA translated into functional effects on neuronal differentiation, neurite outgrowth was evaluated in WT and S1R knockout (KO) N2a cells under low‐serum differentiation conditions. Although subsequent mechanistic studies focused on NBCA because it demonstrated superior neuroprotective efficacy in oxidative stress models, NBBA was also evaluated in the neurite outgrowth assay to determine whether neurite‐promoting activity was a broader property of N‐benzylamide compounds or specific to NBCA. PRE084 (5 µM) and the S1R antagonist BD1047 (5 µM) were included as pharmacological controls to investigate the contribution of S1R signalling to neurite outgrowth.

**Figure 9:**
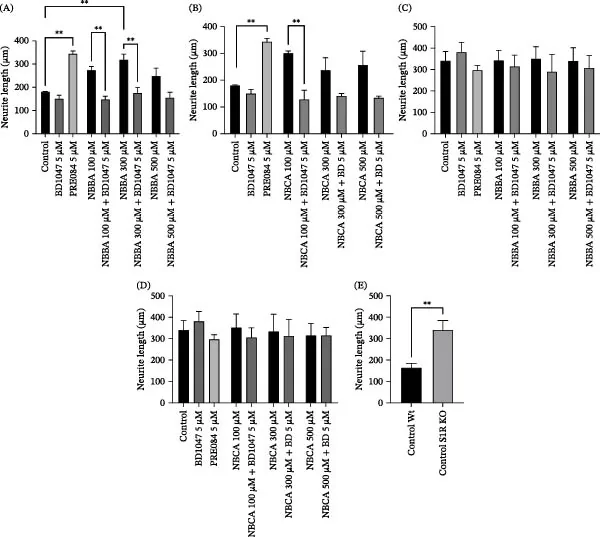
Analysis of N2a cell neurite outgrowth in response to serum starvation and sigma‐1 receptor agonists/antagonists in WT and sigma‐1 receptor KO cells. Data represented as mean ± SEM. (A) Neurite outgrowth in the presence of NBBA in WT N2a cells. (B) Neurite outgrowth in the presence of NBCA in WT N2a cells. (C) Neurite outgrowth in the presence of NBBA in S1R KO N2a cells. (D) Neurite outgrowth in the presence of NBCA in S1R‐KO‐N2a cells. (E) Comparison of WT and S1R KO N2a cell neurite outgrowth. ANOVA followed by Tukey’s statistical test of significance  ^∗∗^ *p*  < 0.01 (*n* = 3).

In WT Neuro‐2a cells, PRE084 significantly increased neurite length compared with untreated controls, whereas BD1047 significantly reduced neurite outgrowth (Figure 9A). NBBA treatment significantly increased neurite length at 100 and 300 μM. However, co‐treatment with BD1047 abolished NBBA’s neurite‐promoting effects at both concentrations. In contrast, 500 μM NBBA did not significantly increase the neurite length compared with untreated controls.

NBCA similarly promoted neurite outgrowth in WT Neuro‐2a cells (Figure 9B). Significant increases in neurite length were observed following treatment with 100 and 300 μM NBCA compared with untreated controls. Co‐treatment with BD1047 significantly reduced neurite length compared with NBCA treatment alone, indicating the attenuation of neurite‐promoting effects. Treatment with 500 μM NBCA did not significantly increase the neurite length.

In S1R knockout Neuro‐2a cells, neither PRE084 nor NBBA significantly altered neurite length compared with untreated knockout controls (Figure 9C). Likewise, co‐treatment with BD1047 did not significantly affect neurite outgrowth. Similarly, neither NBCA nor PRE084 significantly affected neurite length in S1R knockout cells, and BD1047 did not alter the response to NBCA (Figure 9D).

Interestingly, untreated S1R knockout Neuro‐2a cells exhibited significantly longer neurites than untreated WT cells (Figure 9E). This difference was observed under identical differentiation conditions in the absence of pharmacological treatment, indicating that the loss of S1R is associated with altered basal neurite morphology. Representative phase contrast images illustrating neurite morphology for each treatment group are shown in Supporting Information 1: Figures S8–S11.

### 3.7. Molecular Docking Predicts Favourable Interactions of NBCA With S1R, TMEM97 and BiP

**Figure 10:**
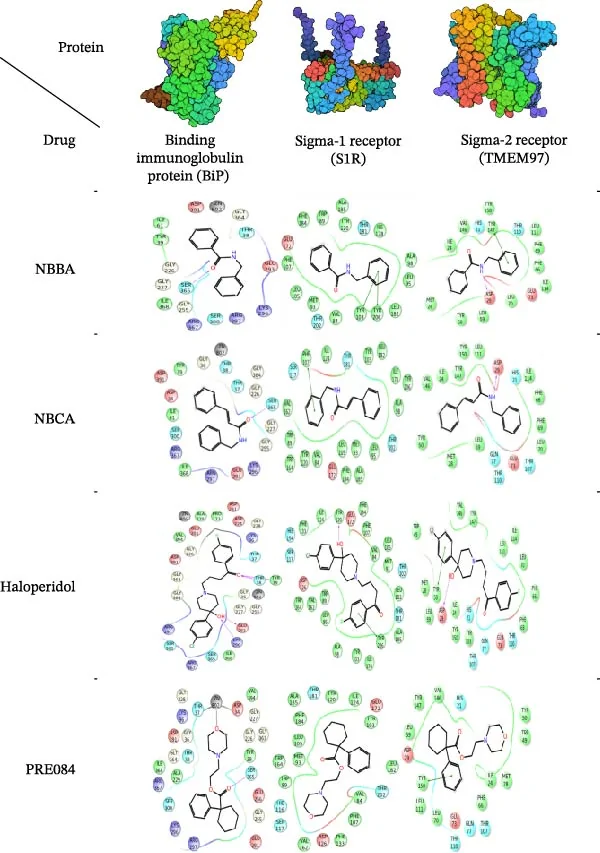
Intermolecular interactions of the NBBA, NBCA, Haloperidol and PRE084 in the active site of binding immunoglobulin protein (BiP), sigma receptor‐1 (S1R) protein and sigma receptor‐2 (S2R/TMEM97).

To investigate the molecular targets that may contribute to the neuroprotective effects of NBCA, molecular docking analyses were performed using three proteins implicated in cellular stress responses and sigma receptor pharmacology. S1R was selected as the primary target because pharmacological and immunofluorescence studies indicated its involvement in NBCA’s biological actions. Binding immunoglobulin protein (BiP/GRP78) was included because it is the principal ER chaperone that directly associates with S1R to regulate its activity under physiological and cellular stress conditions. TMEM97 (sigma‐2 receptor) was also evaluated because several neuroactive benzamide derivatives have been reported to interact with sigma receptor family members, raising the possibility that TMEM97 could contribute to the observed biological responses. Representative three‐dimensional binding poses and two‐dimensional interaction maps are presented in Figure 10, while the complete docking parameters, predicted binding energies and interacting amino acid residues are provided in Supporting Information 2: Tables S1–S6.

Among the compounds evaluated, NBCA demonstrated the most favourable predicted binding affinity for both S1R (−7.97 kcal/mol) and TMEM97 (−10.10 kcal/mol) while also exhibiting strong predicted binding to BiP (−9.58 kcal/mol). In comparison, NBBA displayed weaker predicted binding to S1R (−4.55 kcal/mol) but comparable predicted interactions with BiP (−9.13 kcal/mol) and TMEM97 (−5.50 kcal/mol). The reference ligands PRE084 and haloperidol also demonstrated predicted interactions with all three target proteins, consistent with their established pharmacological activities.

Inspection of the predicted binding poses revealed that NBCA and NBBA occupied the established ligand‐binding pocket of S1R in orientations comparable to those of PRE084 and haloperidol (Figure 10). NBCA shared several conserved interactions with the reference ligands, including residues surrounding the canonical S1R binding site, such as Glu172, Phe107, Thr181, Tyr103 and Trp89, and formed additional contacts with Val162 and Trp164 that may contribute to binding stability (Supporting Information 2: Table S5). A similar overlap in interacting residues was observed for TMEM97 and BiP, where all four compounds were predicted to occupy common binding regions within each protein (Supporting Information 2: Tables S4 and S6).

Overall, the molecular docking analyses predicted that NBCA can interact with S1R, TMEM97 and BiP via binding modes comparable to those of established sigma receptor ligands. The favourable predicted interaction of NBCA with S1R is consistent with the pharmacological inhibition studies, S1R knockout experiments, and immunofluorescence analyses presented in this study, supporting S1R as a principal molecular target underlying the observed neuroprotective effects.

### 3.8. NBCA and NBBA Exhibit Limited Effects on Lifespan in *C. elegans* Models

Having established the neuroprotective activity and S1R‐associated mechanisms of NBCA in cellular models, we next investigated whether these effects translated to whole‐organism models. Lifespan analyses were performed in WT (N2) and amyloid‐β‐overexpressing CL2006 *C*. *elegans* to determine whether chronic exposure to NBCA or NBBA influenced organismal survival under physiological and neurodegenerative conditions.

**Figure 11:**
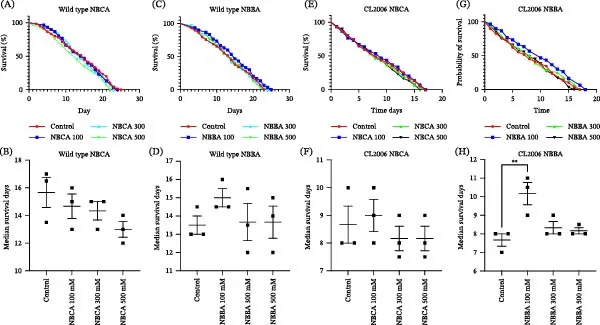
Kaplan–Meier curves and median lifespans. Data represented as mean ± SEM. (A,B) NBCA in wild‐type worms, (C,D) NBBA in wild‐type worms, (E,F) NBCA in transgenic Aβ overexpressing CL2006 strain, (G,H) NBBA in transgenic Aβ overexpressing CL2006 strain. Statistical analysis was carried out using ANOVA followed by Dunnett’s *post hoc* test for statistical significance.  ^∗∗^(*p*  < 0.01). (*n* = 3).

Kaplan–Meier survival analysis demonstrated that treatment with NBCA had no significant effect on the lifespan of WT *C. elegans* at concentrations of 100, 300 or 500 nM compared with vehicle‐treated controls (Figure 11A). Consistent with these observations, median survival was not significantly altered by NBCA treatment in WT worms (Figure 11B).

Similarly, NBBA treatment did not significantly influence the lifespan of WT worms at any concentration tested (Figure 11C,D).

In the transgenic amyloid‐β‐overexpressing CL2006 strain, NBCA likewise produced no significant changes in either the Kaplan–Meier survival curves or the median lifespan compared with untreated animals (Figure 11E,F).

In contrast, treatment of CL2006 worms with 100 nM NBBA produced a modest but significant increase in median lifespan compared with vehicle‐treated animals, whereas the higher concentrations (300 and 500 nM) did not significantly affect survival (Figure 11G,H).

### 3.9. NBCA Improves Survival Following Oxidative Stress in *C. elegans*

Because NBCA exhibited robust antioxidant and cytoprotective activity in cellular models, we next investigated whether these effects translated into improved organismal resistance to oxidative stress. WT *C. elegans* were challenged with H_2_O_2_ (200 µM) in the presence or absence of NBCA, PRE084, haloperidol or their combination, and survival was assessed by Kaplan–Meier analysis and median lifespan.

**Figure 12:**
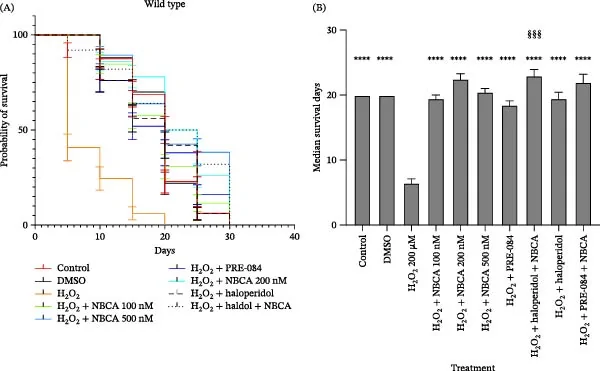
The effect of NBCA on wild‐type worm survival after H_2_O_2_ insult. (A) Kaplan–Meier curves and (B) median lifespans for NBCA in wild‐type worms treated with 200 µM H_2_O_2_. Data presented as mean ± SEM (*n* = 3). Statistical analysis carried out using ANOVA followed by Tukey’s *post hoc* test for statistical significance. Versus control  ^∗^(*p*  < 0.05),  ^∗∗∗∗^(*p*  < 0.0001), versus H_2_O_2_ + PRE084 §§§(*p*  < 0.001).

Exposure to H_2_O_2_ markedly reduced worm survival compared with untreated controls (Figure 12A,B). Treatment with NBCA significantly attenuated this reduction in a concentration‐dependent manner, with 100, 200 and 500 nM NBCA all significantly increasing median survival compared with the H_2_O_2_‐treated group. The greatest protective effect was observed at 200 nM NBCA, which restored the survival to a level comparable with untreated control worms (Figure 12B).

Treatment with the S1R agonist PRE084 also significantly improved survival following H_2_O_2_ exposure, although the increase in median lifespan was less pronounced than that observed with 200 nM NBCA. Co‐treatment with PRE084 and NBCA further improved survival compared with H_2_O_2_ treatment alone and produced the highest median lifespan among the treatment groups, indicating that both compounds effectively protected worms against oxidative stress‐induced mortality.

### 3.10. NBCA Attenuates Oxidative Stress‐Induced Behavioural Deficits Through an S1R‐Dependent Mechanism

To determine whether the cellular neuroprotective effects of NBCA translated into improved neuronal function, we next evaluated naïve chemotaxis, associative learning and short‐term associative memory in WT and TM3443 (S1R ortholog knockout) *C. elegans* following chronic oxidative stress.

**Figure 13:**
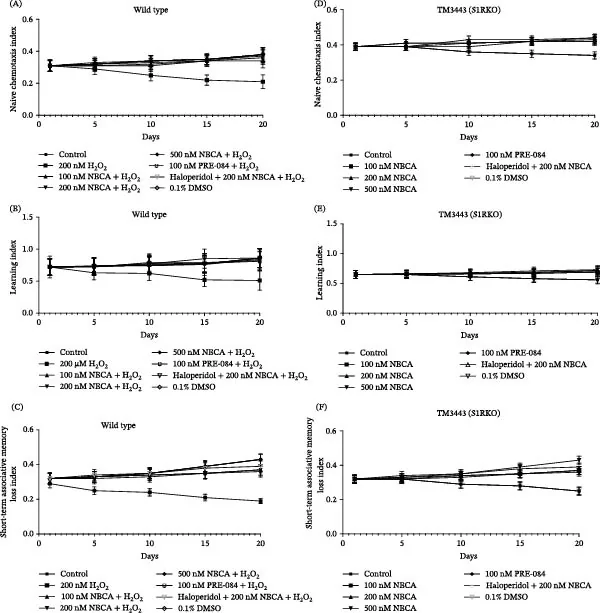
Effect of NBCA on naive chemotaxis, learning index and short‐term associative memory loss index in H_2_O_2_‐treated wild‐type and TM3443 (S1RKO) strains of *C. elegans* for 30 days. (A) wild‐type naive chemotaxis assay. (B) wild‐type learning index. (C) Wild‐type short‐term associative memory loss index. (D) TM3443 naive chemotaxis assay. (E) TM3443 learning index. (F) TM3443 short‐term associative memory loss index.

In WT worms, chronic H_2_O_2_ exposure progressively impaired naïve chemotaxis, learning and short‐term associative memory over the 30‐day experimental period (Figure 13A–C). Treatment with NBCA significantly attenuated these behavioural deficits, with all three concentrations (100, 200 and 500 nM) preserving chemotaxis, learning performance and short‐term associative memory compared with H_2_O_2_‐treated worms. The protective effects of NBCA were comparable to those observed with the selective S1R agonist PRE084. Co‐treatment with PRE084 and NBCA did not produce an obvious improvement beyond either treatment alone, whereas co‐treatment with the S1R antagonist, haloperidol, abolished the behavioural benefits of NBCA (Figure 13A–C).

To further investigate the involvement of S1R, the same behavioural assays were performed in the TM3443 S1R knockout strain (Figure 13D–F). In contrast to WT worms, NBCA failed to preserve naïve chemotaxis, learning or short‐term associative memory in TM3443 animals following H_2_O_2_ exposure. Similarly, PRE084 did not improve the behavioural performance in the knockout strain, and co‐treatment with haloperidol produced no additional effect. These findings demonstrate that the behavioural protection afforded by NBCA under oxidative stress is dependent upon the presence of functional S1R.

### 3.11. NBCA Preserves Behavioural Performance in AD Models of *C. elegans*

Having demonstrated that NBCA protected against oxidative stress‐induced behavioural deficits in WT worms, we next investigated whether these neuroprotective effects extended to transgenic *C. elegans* models of AD. Behavioural performance was therefore assessed in the Aβ‐overexpressing CL2006 and CL4176 strains by measuring naïve chemotaxis, associative learning and short‐term associative memory over a 20‐day treatment period.

**Figure 14:**
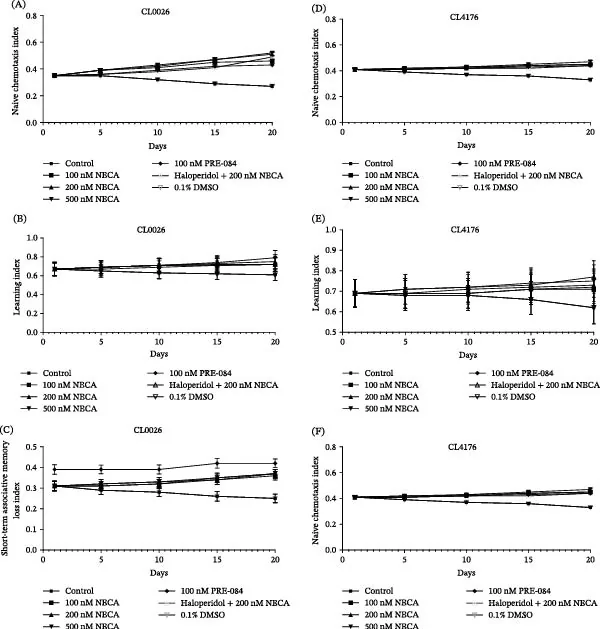
Effect of NBCA on naive chemotaxis, learning index and short‐term associative memory loss index in Aβ‐overexpressing CL2006 and CL4176 strains of *C. elegans* for 20 days. (A) CL2006 naive chemotaxis assay. (B) CL2006 learning index. (C) CL2006 short‐term associative memory loss index. (D) CL4176 naive chemotaxis assay. (E) CL4176 learning index. (F) CL4176 short‐term associative memory loss index.

In the constitutively Aβ‐overexpressing CL2006 strain, untreated animals exhibited progressive impairments in naïve chemotaxis, learning and short‐term associative memory throughout the experimental period (Figure 14A–C). Treatment with NBCA significantly improved behavioural performance in all three assays, with the 100, 200 and 500 nM concentrations producing similar protective effects. PRE084 likewise improved behavioural performance, whereas co‐treatment with the S1R antagonist haloperidol attenuated the beneficial effects of NBCA, supporting the involvement of S1R signalling in mediating these responses.

Comparable findings were obtained in the temperature‐inducible Aβ‐overexpressing CL4176 strain (Figure 14D–F). NBCA significantly preserved naïve chemotaxis, learning and short‐term associative memory relative to untreated animals, with behavioural performance approaching that observed in healthy control worms. PRE084 produced similar improvements, while haloperidol diminished the protective effects of NBCA. Collectively, these findings demonstrate that NBCA preserves neuronal function in two independent *C. elegans* models of AD and further support an S1R‐associated mechanism underlying its neuroprotective activity.

### 3.12. NBCA and NBBA Attenuate Aβ‐Induced Paralysis in Transgenic *C. elegans*

**Figure 15:**
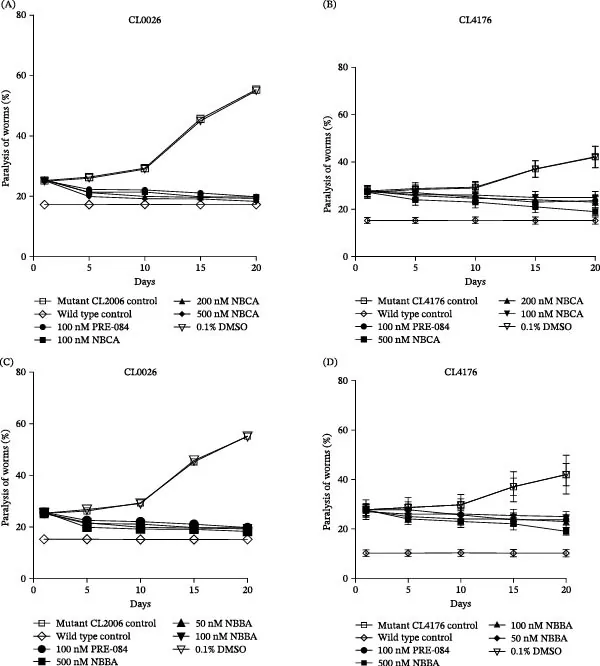
Effect of NBCA and NBBA on Aβ‐induced paralysis in transgenic *C. elegans* over 20 days. Paralysis was assessed in the CL2006 and CL4176 strains following a 36 h treatment period. (A) CL2006 worms treated with NBCA. (B) CL4176 worms treated with NBCA. (C) CL2006 worms treated with NBBA. (D) CL4176 worms treated with NBBA. At each time point, only living adult worms were scored; larvae, visibly smaller progeny and dead worms were excluded. Data are presented as the percentage of living adult worms exhibiting paralysis (mean ± SEM, *n* = 3).

To determine whether the behavioural improvements observed in the AD models were accompanied by reduced disease pathology, the effects of NBCA and NBBA on the development of paralysis were examined in the Aβ‐overexpressing CL2006 and CL4176 strains over a 20‐day observation period (Figure 15).

In the constitutively expressing CL2006 strain, untreated transgenic worms exhibited a progressive increase in paralysis, reaching ~55% by day 20, whereas WT animals showed minimal paralysis throughout the experiment (Figure 15A). Treatment with NBCA markedly attenuated the development of paralysis at all concentrations tested, maintaining paralysis levels close to those observed in WT controls. A similar protective effect was observed following treatment with the reference S1R agonist PRE084.

Comparable findings were obtained in the inducible CL4176 strain, in which untreated transgenic worms developed progressively increasing paralysis over a 20‐day observation period (Figure 15B). NBCA treatment substantially reduced paralysis in all treatment groups, again producing effects comparable with PRE084.

NBBA also reduced the development of paralysis in both CL2006 and CL4176 worms (Figure 15C,D). Although all concentrations produced protective effects relative to untreated transgenic animals, the magnitude of protection was generally less pronounced than that observed with NBCA. Collectively, these findings demonstrate that both N‐benzylamide compounds attenuate Aβ‐associated paralysis in vivo, with NBCA exhibiting a greater overall protective effect.

### 3.13. NBCA Reduces Amyloid‐β Accumulation in Transgenic *C. elegans*

**Figure 16:**
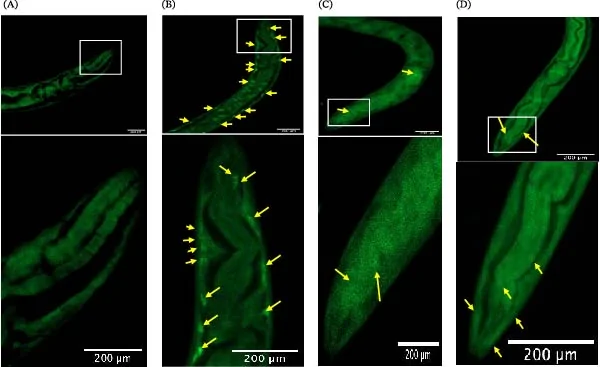
Thioflavin S staining of amyloid deposition in *C. elegans*. (A) Wild‐type N2 worms showing minimal Thioflavin S‐positive staining. (B) Untreated Aβ‐overexpressing CL2006 worms demonstrating abundant Thioflavin S‐positive deposits (yellow arrows). (C) CL2006 worms treated with 100 nM PRE084 showing reduced amyloid deposition. (D) CL2006 worms treated with 500 nM NBCA showing reduced Thioflavin S‐positive deposits. White boxes indicate the enlarged head region shown below each corresponding image. Scale bars = 200 μm.

To determine whether the behavioural improvements observed in the transgenic AD models were associated with reduced amyloid‐β pathology, amyloid deposition was examined in CL2006 worms using Thioflavin S staining (Figure 16).

WT N2 worms exhibited minimal Thioflavin S‐immunoreactivity, with little evidence of amyloid‐positive deposits within the head region (Figure 16A). In contrast, untreated CL2006 worms displayed numerous discrete Thioflavin S‐positive deposits distributed throughout the body and head region, consistent with the accumulation of amyloid‐β aggregates (Figure 16B).

Treatment with the reference S1R agonist PRE084 reduced the abundance and intensity of Thioflavin S‐positive deposits compared with untreated CL2006 worms (Figure 16C). Similarly, treatment with 500 nM NBCA markedly reduced the number and apparent intensity of amyloid‐positive deposits throughout the animal, including within the enlarged head region shown in the lower panel (Figure 16D). Collectively, these observations indicate that NBCA attenuates amyloid‐β accumulation in vivo, consistent with improvements in behavioural performance in transgenic AD models.

## 4. Discussion

The present study demonstrates that NBCA exerts broad neuroprotective effects across complementary in vitro, in vivo and in silico models of neurodegeneration. NBCA protected neuronal cells against glutamate‐ and H_2_O_2_‐induced oxidative injury, attenuated intracellular oxidative stress, restored endogenous antioxidant defences and preserved cholinergic enzyme activity. Furthermore, NBCA modulated S1R‐ and NRF2‐associated stress responses, promoted neurite outgrowth through S1R‐dependent mechanisms, improved behavioural performance in multiple *C. elegans* models and reduced amyloid‐β accumulation in vivo. Although the structurally related analogue NBBA demonstrated neuroprotective activity in selected assays, its effects were generally less pronounced than those of NBCA. Collectively, these findings identify NBCA as a promising neuroprotective scaffold that acts by preserving cellular stress homeostasis and modulating S1R‐associated signalling pathways.

Oxidative stress and mitochondrial dysfunction are central pathological mechanisms underlying many neurodegenerative disorders, including AD, PD and ALS [32]. Consequently, compounds capable of preserving neuronal redox homeostasis represent attractive therapeutic candidates. In the present study, NBCA protected neuronal cells against both glutamate‐ and H_2_O_2_‐induced oxidative injury, two complementary models that reproduce distinct aspects of oxidative stress. HT22 cells undergo glutathione depletion and ROS accumulation following glutamate exposure [33], whereas H_2_O_2_ directly generates oxidative stress, mitochondrial dysfunction and lipid peroxidation [34]. The ability of NBCA to confer protection in both models suggests that its neuroprotective activity is not restricted to a single mechanism of oxidative injury. Although glutamate toxicity can also be investigated in SH‐SY5Y cells and H_2_O_2_ in HT22 cells, the present study employed the most widely established oxidative stress paradigm for each cell line to maximise biological relevance and facilitate comparison with previous studies [35].

The antioxidant effects of NBCA were accompanied by alterations in NRF2‐associated stress responses. NRF2 is a master regulator of cellular antioxidant defence that is activated in response to oxidative stress, promoting the transcription of genes involved in glutathione metabolism, ROS detoxification and cellular redox homeostasis [36]. Consistent with previous studies, H_2_O_2_ exposure increased NRF2 immunoreactivity, reflecting the activation of endogenous antioxidant defence mechanisms in response to oxidative injury [37, 38]. Interestingly, co‐treatment with NBCA significantly attenuated this increase, with the greatest effect observed at 500 nM. PRE084 produced a similar trend, although the reduction in NRF2 immunoreactivity did not reach statistical significance.

At first consideration, a reduction in NRF2 immunoreactivity might appear inconsistent with a neuroprotective mechanism. However, when interpreted alongside the marked reductions in intracellular ROS, lipid peroxidation and restoration of endogenous antioxidant enzyme activities observed following NBCA treatment, the findings more likely reflect a reduced requirement for compensatory activation of NRF2 signalling. In other words, by limiting oxidative stress at an earlier stage, NBCA may reduce the cellular demand for sustained NRF2 activation. An alternative, but not mutually exclusive, explanation is that NBCA directly influences components of the S1R–NRF2 signalling axis. Previous studies have demonstrated that S1R activation can facilitate NRF2‐dependent antioxidant responses by regulating ER stress and MAM signalling [39]. However, the present study measured total NRF2 immunoreactivity rather than nuclear translocation or transcriptional activity and therefore cannot distinguish between reduced pathway activation secondary to diminished oxidative stress and direct modulation of NRF2 signalling. Future studies examining NRF2 nuclear localisation, activation of the antioxidant response element, and downstream target gene expression will be required to clarify the molecular relationship among NBCA, S1R and NRF2 signalling.

Oxidative stress contributes not only to neuronal injury but also to the disruption of cholinergic neurotransmission [40, 41], a hallmark of AD closely associated with progressive cognitive decline [42]. Excessive oxidative stress has been shown to reduce acetylcholine availability by altering AChE and BuChE activity, thereby impairing synaptic transmission and cognitive function [43]. In the present study, H_2_O_2_ markedly decreased AChE activity while simultaneously increasing BuChE activity, changes consistent with oxidative stress‐induced disruption of cholinergic homeostasis. NBCA effectively restored AChE activity and normalised BuChE activity towards control levels, suggesting that its neuroprotective effects extend beyond the preservation of cell viability to maintenance of neurotransmitter regulatory pathways.

The restoration of cholinergic enzyme activity is likely to result, at least in part, from the reduction in oxidative stress achieved by NBCA as oxidative damage is known to influence both cholinesterase activity and cholinergic neurone function. PRE084 produced similar protective effects, whereas haloperidol attenuated NBCA’s actions, further supporting the involvement of S1R‐associated signalling. Although the present study did not directly measure acetylcholine concentrations or synaptic transmission, preservation of cholinergic enzyme homeostasis provides an additional mechanism through which NBCA may contribute to improved neuronal function and cognitive performance. This interpretation is further supported by the behavioural improvements observed in the *C. elegans* models of oxidative stress and AD described later in this study.

The present study provides several independent lines of evidence supporting the involvement of S1R‐associated signalling in the neuroprotective actions of NBCA. S1R is an ER‐resident ligand‐operated chaperone that is enriched at mitochondria‐associated ER membranes (MAMs), where it regulates calcium homeostasis, mitochondrial function, ER stress responses and cellular adaptation to physiological stress [44–46]. Under basal conditions, S1R forms a regulatory complex with the ER chaperone binding immunoglobulin protein (BiP/GRP78) [47]. Cellular stress or pharmacological modulation promotes dissociation of this complex, allowing S1R to interact with multiple client proteins and activate signalling pathways involved in neuronal survival, mitochondrial function and cellular resilience [48, 49].

Consistent with this established role, H_2_O_2_ treatment significantly increased S1R immunoreactivity, likely reflecting the activation of endogenous stress‐response mechanisms following oxidative injury [19]. Co‐treatment with NBCA attenuated this response, producing a pattern similar to that observed for NRF2. When considered alongside the marked reductions in ROS production, lipid peroxidation and restoration of antioxidant enzyme activities, these findings suggest that NBCA reduces the cellular stress burden, thereby decreasing the requirement for compensatory S1R activation. A similar trend was observed following treatment with the reference S1R agonist PRE084, whereas haloperidol attenuated the protective effects of NBCA across multiple experimental models. Although changes in total S1R immunoreactivity cannot establish receptor activation or ligand binding, the pharmacological profile observed throughout this study is consistent with the modulation of S1R‐associated cellular stress signalling.

Unlike studies that rely solely on pharmacological inhibitors or receptor expression analyses, the present work combines multiple complementary approaches, including pharmacological modulation, immunofluorescence, genetic knockout models and computational docking. While no single experiment independently demonstrates direct receptor engagement, the convergence of these findings provides stronger evidence that S1R‐associated pathways contribute to the biological activity of NBCA than any individual assay alone. Nevertheless, direct receptor‐binding studies and functional characterisation of S1R activation will be required to determine whether NBCA acts as an orthosteric ligand, an allosteric modulator, or indirectly influences S1R signalling by regulating upstream cellular stress pathways.

Among the mechanistic findings presented in this study, the neurite outgrowth experiments provide the strongest functional evidence supporting the involvement of S1R‐associated signalling in the biological activity of NBCA. Promotion of neurite extension is a well‐established downstream consequence of S1R activation and has been reported for a diverse range of structurally distinct S1R ligands and positive modulators [50–52]. Consistent with these observations, both NBCA and NBBA significantly promoted neurite outgrowth in WT Neuro‐2a cells. Importantly, these effects were markedly attenuated by the selective S1R antagonist BD1047 and were completely abolished in S1R knockout cells, demonstrating that receptor expression is required for the neuritogenic effects of both compounds.

These findings substantially strengthen the mechanistic conclusions of the present study by extending beyond correlative changes in protein immunoreactivity or pharmacological inhibition alone. While the oxidative stress experiments demonstrated that NBCA attenuated cellular injury and modulated S1R‐associated stress responses, the combined pharmacological and genetic evidence obtained from the neurite outgrowth assays provides direct functional support for the involvement of S1R‐associated signalling. Nevertheless, the present findings do not establish whether NBCA acts directly via S1R or indirectly by modulating upstream cellular stress pathways that converge on S1R‐dependent signalling.

An unexpected finding of the present study was that untreated S1R knockout Neuro‐2a cells exhibited significantly greater basal neurite outgrowth than their WT counterparts. At first glance, this observation appears paradoxical because pharmacological activation of S1R has consistently been associated with enhanced neurite extension, neuronal differentiation and synaptic plasticity. However, despite the increased basal neurite length observed in the knockout cells, both NBCA and NBBA completely lost their neuritogenic activity in the absence of S1R. These findings suggest that the role of S1R in regulating neurite dynamics is more complex than that of a simple positive mediator of neurite extension.

One possible explanation is that chronic deletion of S1R results in compensatory cellular adaptations that alter pathways controlling cytoskeletal organisation or neuronal differentiation. Alternatively, disruption of the constitutive S1R–BiP complex may modify ER stress signalling or BiP availability for interactions with other client proteins involved in neurite development. Although these possibilities remain speculative, they highlight the dynamic role of S1R within broader cellular stress‐response networks rather than functioning solely as an isolated receptor. Future studies examining BiP function, S1R–BiP complex formation and downstream signalling pathways will be required to determine the molecular basis of this previously unrecognised phenotype.

To complement the experimental findings, molecular docking analyses were performed to investigate whether NBCA could plausibly interact with proteins implicated in the observed neuroprotective responses. S1R was selected because the pharmacological, immunofluorescence and genetic studies consistently implicated S1R‐associated signalling in the biological activity of NBCA. BiP was included because it is the principal ER chaperone that forms a regulatory complex with S1R under resting conditions and plays a central role in coordinating cellular stress responses [27]. TMEM97 was also examined because several neuroactive benzamide derivatives have been reported to interact with sigma receptor family members, suggesting that additional sigma receptor‐associated pathways may contribute to the biological activity of NBCA.

Docking analyses predicted favourable interactions between NBCA and all three target proteins, with the strongest predicted affinity observed for TMEM97, followed by BiP and S1R. Within the S1R binding pocket, NBCA adopted a binding orientation comparable to that of the established ligands PRE084 and haloperidol and shared several conserved interactions with residues known to contribute to ligand recognition. While these findings are consistent with the experimental evidence supporting S1R‐associated signalling, molecular docking alone cannot establish direct receptor binding or pharmacological activity as direct receptor binding and pharmacological characterisation remain to be established.

Particularly intriguing was the predicted interaction between NBCA and BiP. The S1R–BiP complex is increasingly recognised as a central regulator of cellular adaptation to oxidative and ER stress, with dissociation of the complex facilitating activation of S1R‐dependent cytoprotective signalling. Consequently, the ability of NBCA to dock favourably with both S1R and BiP raises the possibility that the compound may influence this regulatory complex rather than acting exclusively through direct interaction with S1R. Although this hypothesis is attractive and is consistent with the enhanced basal neurite outgrowth observed in S1R‐deficient cells, the present study did not directly examine S1R–BiP interactions or BiP function. Therefore, the proposed involvement of BiP should be considered speculative and requires experimental validation through approaches such as co‐immunoprecipitation, proximity ligation assays and functional studies examining S1R–BiP complex dynamics.

The behavioural studies performed in *C. elegans* demonstrate that the neuroprotective effects of NBCA extend beyond cultured neuronal cells and translate into preservation of neuronal function in vivo. Importantly, NBCA did not significantly alter lifespan under basal conditions, indicating that its biological effects are unlikely to reflect a general extension of longevity. In contrast, NBCA significantly improved survival following H_2_O_2_‐induced oxidative stress, consistent with its antioxidant and cytoprotective actions observed in the mammalian cell models. Together, these findings suggest that NBCA enhances resistance to cellular stress rather than acting as a non‐specific longevity‐promoting compound.

This protective effect was further reflected in behavioural performance. Chronic oxidative stress impaired naïve chemotaxis, associative learning and short‐term memory in WT worms, whereas NBCA significantly preserved all three behavioural endpoints. PRE084 produced comparable improvements, while haloperidol attenuated the protective effects of NBCA, supporting the involvement of S1R‐associated signalling. These observations are consistent with previous reports demonstrating that pharmacological modulation of S1R can improve learning, memory and neuronal resilience in models of ageing, neurodegeneration and central nervous system injury. The ability of NBCA to preserve behavioural performance therefore provides important functional validation of the cellular mechanisms identified in the in vitro studies.

The neuroprotective effects of NBCA were not restricted to oxidative stress but also extended to transgenic *C. elegans* models of AD. In both the constitutive Aβ‐overexpressing CL2006 strain and the inducible CL4176 strain, NBCA significantly improved naïve chemotaxis, learning and short‐term associative memory. These functional improvements were accompanied by attenuation of Aβ‐induced paralysis and a reduction in Thioflavin S‐positive amyloid deposits, indicating that behavioural preservation was associated with reduced pathological burden. Collectively, these findings demonstrate that the cellular protection afforded by NBCA translates into meaningful improvements in neuronal function and pathology within whole‐organism models of neurodegeneration.

Although the *C. elegans* models do not fully recapitulate the complexity of human AD, they provide an established platform for evaluating conserved pathways regulating oxidative stress, proteostasis and neuronal function. Consequently, the concordance between the cellular and organismal findings substantially strengthens the evidence supporting the neuroprotective potential of NBCA.

One particularly interesting finding emerged from studies using the TM3443 strain lacking the *C. elegans* S1R orthologue. In contrast to the Neuro‐2a knockout experiments, where deletion of S1R abolished the neuritogenic effects of NBCA, beneficial effects on chemotaxis, learning and memory were still observed in TM3443 worms. These findings suggest that the behavioural actions of NBCA cannot be explained solely by signalling through the canonical S1R orthologue and instead indicate that additional molecular targets or compensatory signalling pathways may contribute to its neuroprotective activity in vivo.

Several explanations may account for this apparent discrepancy. Chronic genetic deletion of S1R may induce compensatory adaptations that enhance the contribution of parallel stress‐response pathways during ageing. Alternatively, NBCA may influence additional targets involved in regulating oxidative stress, mitochondrial function or proteostasis. The favourable docking of NBCA to TMEM97 raises the possibility that sigma receptor family members other than S1R contribute to the behavioural responses observed in *C. elegans*, although this remains speculative because TMEM97 signalling was not examined experimentally in the present study. Regardless of the underlying mechanism, these findings suggest that NBCA engages a broader network of cytoprotective pathways than S1R alone, consistent with its ability to preserve the neuronal function across multiple experimental models.

Although the present study provides evidence supporting the neuroprotective potential of NBCA across multiple experimental models, several limitations should be acknowledged. First, while the combined pharmacological, genetic and computational data strongly support the involvement of S1R‐associated signalling, direct receptor binding was not experimentally evaluated. Consequently, it remains unclear whether NBCA functions as a direct S1R ligand, an allosteric modulator, or indirectly influences S1R signalling by modulating upstream cellular stress pathways. Future studies employing radioligand binding assays, receptor occupancy measurements, and pharmacological characterisation will be required to more precisely define the interaction between NBCA and S1R.

Second, although molecular docking predicted favourable interactions between NBCA and BiP, and the enhanced basal neurite outgrowth observed in S1R knockout cells is consistent with altered S1R–BiP signalling, the present study did not directly examine BiP expression, localisation or S1R–BiP complex dynamics. Consequently, the proposed role of BiP should be regarded as a mechanistic hypothesis that requires experimental validation. Likewise, while favourable docking to TMEM97 suggests that additional sigma receptor‐associated pathways may contribute to the biological activity of NBCA, this possibility was not investigated experimentally and therefore remains speculative.

Finally, the present study focused primarily on defining the neuroprotective mechanisms of NBCA in cellular models and validating these findings in *C. elegans*. Although these complementary models provide robust evidence supporting the biological activity of NBCA, further studies using mammalian models of neurodegeneration will be required to establish its pharmacokinetic properties, brain exposure, long‐term efficacy and therapeutic potential in vivo. Such studies will be essential to determine whether the mechanisms identified here translate to more clinically relevant models of NDD.

## 5. Conclusion

In conclusion, the present study demonstrates that NBCA exerts robust neuroprotective effects across complementary cellular, organismal and computational models of neurodegeneration. NBCA protected neurones from oxidative injury by preserving redox homeostasis, maintaining mitochondrial function, restoring endogenous antioxidant defences and preserving cholinergic enzyme activity. These cellular effects were accompanied by modulation of S1R‐associated stress responses, promotion of neurite outgrowth and preservation of neuronal function in multiple *C. elegans* models of oxidative stress and AD, together with reduced amyloid‐β accumulation and delayed paralysis.

Collectively, the pharmacological, genetic and computational evidence supports the involvement of S1R‐associated signalling in NBCA’s biological activity and suggests that additional cellular stress‐response pathways may also contribute to its neuroprotective actions. Although further studies are required to define its direct molecular targets and pharmacological properties, the present findings identify NBCA as a promising neuroprotective scaffold and provide a strong foundation for the future development of N‐benzylamide derivatives as therapeutic candidates for AD and related neurodegenerative disorders.

## Author Contributions

**Subaraja Mamangam:** writing – review & editing, writing – original draft, methodology, investigation, formal analysis. **Anantha Krishnan Dhanabalan**: methodology, investigation, formal analysis, Data curation. **Kanika Verma**: methodology, investigation, formal analysis, data curation. **Mani Iyer Prasanth**: investigation, formal analysis. **Sirikalaya Brimson**: writing – review and editing, project administration, and funding acquisition. **James Michael Brimson**: writing – review and editing, writing – original draft, supervision, project administration, funding acquisition, conceptualisation.

## Funding

This study was supported by the National Research Council of Thailand (Grant N42A670287) and by the Ratchansaphiseksomphot fund for the development of new faculty staff Chulalongkorn University, DNS 68 002 3700 001.

## Conflicts of Interest

James M. Brimson reports that financial support was provided by the National Research Council of Thailand. All other authors declare no conflicts of interest.

## Supporting Information

Additional supporting information can be found online in the Supporting Information section.

## Supporting information


**Supporting Information 1** Figures S1–S11 showing additional SH‐SY5Y cell viability data, NRF2 and sigma‐1 receptor immunofluorescence images and staining controls, and neurite‐outgrowth analyses in wild‐type and sigma‐1 receptor knockout N2A cells treated with NBBA or NBCA.

**Supporting Information 2** Tables S1–S6 presenting molecular docking parameters and interacting amino‐acid residues for NBBA, NBCA, PRE‐084 and haloperidol with binding immunoglobulin protein (BiP), sigma‐1 receptor (S1R), and sigma‐2 receptor (S2R/TMEM97).

## Data Availability

Available from authors upon reasonable request.
