# Supplementary material for: Neuroprotective Effects of Two N‐Benzylamides in Models of Oxidative Stress, Neurite Outgrowth and Neurodegeneration
Source: Oxid Med Cell Longev. 2026 Sep 17;2026:3092494. doi: 10.1155/omcl/3092494 (PMC13583929; doi:10.1155/omcl/3092494)
Supplement: Supplementary file 1 [file OMCL-2026-3092494-s001.docx]

**Neuroprotective Effects of Two N-Benzylamides in Models of Oxidative Stress, Neurite Outgrowth, and Neurodegeneration**

Subaraja Mamangam ^a^, Anantha Krishnan Dhanabalan^b^, Kanika Verma^c^, Mani Iyer Prasanth^d^, Sirikalaya Brimson^e^, and James Michael Brimson ^a,f ‡^

^a^ Research, Innovation and International Affairs, Faculty of Allied Health Sciences, Chulalongkorn University, Bangkok 10330, Thailand.

^b^ RUBi - Research Unit in Bioinformatics, Department of Biochemistry, Microbiology and

Bioinformatics, Rhodes University, Makhanda, 6139, South Africa.

^c^ Department of Molecular Epidemiology, National Institute of Malaria Research, Delhi, India.

^d^ Division of Molecular Genetics and Cancer, NITTE University Centre for Science Education and Research (NUCSER), Mangaluru, Karnataka, India.

^e^ Department of Clinical Microscopy, Faculty of Allied Health Sciences, Chulalongkorn University, Bangkok 10330, Thailand.

^f^ Center of Excellence on Natural Products for Neuroprotection and Anti-ageing (Neur-Age Nat Chula), Chulalongkorn University, Bangkok, 10330, Thailand.

^‡^Corresponding author:

James Michael Brimson

Phone +66 (0)2 218 1569

Email: [jamesmichael.b@chula.ac.th](mailto:jamesmichael.b@chula.ac.th)

Address: ChulaPhat-1 Building, 154 Rama 1 Road, Pathumwan, Bangkok, 10330, Thailand.

**Supplementary Tables**

**Supplementary Table 1.** The binding affinities, glide ligand efficiency, glide energy, and inhibition constant values of the tested compound with the binding immunoglobulin protein (BiP) from screening analysis.

| **Compound** | **Binding energy (Kcal/mol)** | **Glide ligand efficiency**  **(Kcal/mol)** | **Glide Energy**  **(Kcal/mol)** |
| --- | --- | --- | --- |
| N-Benzoylbenzylamine | -9.13 | -0.36 | -34.13 |
| N-Benzylcinnamamide | -9.58 | -0.37 | -34.17 |
| PRE-084 | -4.64 | -0.27 | -33.67 |
| Haloperidol | -8.90 | -0.34 | -38.59 |

**Supplementary Table 2:** The binding affinities, glide ligand efficiency, glide energy, and inhibition constant of the tested compound with the sigma receptor-1 (S1R) protein from screening analysis.

| **Compound** | **Binding energy (Kcal/mol)** | **Glide ligand efficiency**  **(Kcal/mol)** | **Glide Energy**  **(Kcal/mol)** |
| --- | --- | --- | --- |
| N-Benzoylbenzylamine | -4.55 | -0.38 | -35.35 |
| N-Benzylcinnamamide | -7.97 | -0.32 | -39.94 |
| PRE-084 | -6.17 | -0.33 | -25.56 |
| Haloperidol | -4.27 | -0.16 | -43.35 |

**Supplementary Table 3:** The binding affinities, glide ligand efficiency, glide energy, and inhibition constant values of the tested compound with sigma receptor-2 (S2R/TMEM97) proteins from screening analysis.

| **List of compounds** | **Binding energy (Kcal/mol)** | **Glide ligand efficiency**  **(Kcal/mol)** | **Glide Energy**  **(Kcal/mol)** |
| --- | --- | --- | --- |
| N-Benzoylbenzylamine | -5.5 | -0.31 | -36.247 |
| N-Benzylcinnamamide | -10.1 | -0.36 | -41.929 |
| PRE-084 | -4.6 | -0.20 | -33.676 |
| Haloperidol | -8.9 | -0.342 | -55.753 |

**Supplementary Table 4:** Amino acid residues of the binding immunoglobulin protein (BiP) involved in interactions with the tested compound.

| **Compound** | **Major interacting amino acids** | **Minor interacting amino acids** |
| --- | --- | --- |
| NBBA | Yly226, Try 39, Ser365, Ile61, Gly255, Arg367, Ser300, Arg297, Lys296, Glu293, Thr381, Gly364 and ASP391 | Gly and Ile368 |
| NBCA | Gly36, Typ39, Asp391, Thr37, Gly255, Glu293, Ser365, Arg297 and Ile368. | Asp34, Ile61, Ser300, Arg367, Gly227, Gly226, Gly364, Thr38, Thr37, Gly36 |
| PRE-084 | Ala229, Thr38, gly36, Thr37, Aps34, Gly226, Tyr39, Ser365, Arg297 and Ser300 | Arg367, Ile368, Gly364, Thr38, Asp39, Lys96, gly228, Val394, Gly227, Gly363, Glu256, Gly225, Glu293 and Lys296 |
| Haloperidol | Gly364, Gly363, Gly226, Thr37, Thr38, Try39, Gly36, Glu293, lys296, Ser365, Arg367, Ser300, and Arg297. | Asp391, Val394, Glu201, Ala229, Pro173, Asp231, Asp224, Gly225, Lys96, Gly227, Gly225 and Ile368 |

**Supplementary Table 5:** Amino acid residues of the Sigma-1 Receptor (S1R) involved in interactions with the tested compound.

| **Compound** | **Major interacting amino acids** | **Minor interacting amino acids** |
| --- | --- | --- |
| NBBA | Glu172, Phe107, Typ103, Typ206, Trp89 and Phe184 | Ala185, Typ120, Thr181, Ile178, Ala98, Ieu95, Ile182, Val84, Thr202, Met93, and Leu105 |
| NBCA | Phe107, Thr181, and Val162 | Ser117, Ile124, Typ103, Leu182, Ile178, Typ206, Ala98, Thr202, Leu95, met93, Ala185, phe184, Leu105, Glu172, Val84, Try120, Trp164 and Trp89 |
| PRE-084 | Val84 and Thr202 | Leu182, Phe107, Phe133, Asp126, Val16, Ser117, His116, Trp89, Trp164, Met93, Ilu105, Phe184, Ala185, Thr181, Tyr120, Ile124, Glu172 and Tyr103 |
| Haloperidol | His154, Ph133, Ile124, Trp120, Glu172, Phe107, Val84, Ieu182, Thr181, Try206 and Asp126 | Ser117, Phe184, Ieu105, Met93, Thr202, Ala185, Ile178, Try103, Ala185, Ile178, Try103, Ala98, Leu95, Trp89, Val162 and Trp164 |

**Supplementary Table 6:** Amino acid residues of the Sigma-2 Receptor (S2R/TMEM97) involved in interactions with the tested compound.

| **Compound** | **Major interacting amino acids** | **Minor interacting amino acids** |
| --- | --- | --- |
| NBBA | Met28, Asp29, Leu70, Thr110 and Ile24 | Val146, His21, Try150, Leu111, Phe69, Phe66, Ieu114, Glu73, Ieu70, Leu99 and Typ50 |
| NBCA | Ile24, Typ147, ASP29, His21, Leu59 and Try50 | Val146, Typ150, Ieu111, Ile144, Phe66, Phe69, Ieu70, Thr107, Glu73, Gln77, Thr110 and Met28 |
| PRE-084 | Val146, Asp29, Ieu70, Glu73, Thr110, Ilu24, Try150 | Typ50, Trp49, Met28, Phe66, Thr107, Gln77, Ieu111, Ieu59, Try147 and His21 |
| Haloperidol | Typ147, His21, Ile24, Asp29, Typ50 and Trp49 | Val146, Ilu11, Ile114, Ieu70, Phe66,  Phe69, Thr110, Glu73, Gln77, Thr107, Try103, Try150, Leu59 and met28 |
