# Supplementary material for: Neuroprotective Effects of Two N‐Benzylamides in Models of Oxidative Stress, Neurite Outgrowth and Neurodegeneration
Source: Oxid Med Cell Longev. 2026 Sep 17;2026:3092494. doi: 10.1155/omcl/3092494 (PMC13583929; doi:10.1155/omcl/3092494)
Supplement: Supplementary file 2 [file OMCL-2026-3092494-s002.docx]

**Neuroprotective Effects of Two N-Benzylamides in Models of Oxidative Stress, Neurite Outgrowth, and Neurodegeneration**

Subaraja Mamangam ^a^, Anantha Krishnan Dhanabalan^b^, Kanika Verma^c^, Mani Iyer Prasanth^d^, Sirikalaya Brimson^e^, and James Michael Brimson ^a,f ‡^

^a^ Research, Innovation and International Affairs, Faculty of Allied Health Sciences, Chulalongkorn University, Bangkok 10330, Thailand.

^b^ RUBi - Research Unit in Bioinformatics, Department of Biochemistry, Microbiology and

Bioinformatics, Rhodes University, Makhanda, 6139, South Africa.

^c^ Department of Molecular Epidemiology, National Institute of Malaria Research, Delhi, India.

^d^ Division of Molecular Genetics and Cancer, NITTE University Centre for Science Education and Research (NUCSER), Mangaluru, Karnataka, India

^e^ Department of Clinical Microscopy, Faculty of Allied Health Sciences, Chulalongkorn University, Bangkok 10330, Thailand.

^f^ Center of Excellence on Natural Products for Neuroprotection and Anti-ageing (Neur-Age Nat Chula), Chulalongkorn University, Bangkok, 10330, Thailand

^‡^Corresponding author:

James Michael Brimson

Phone +66 (0)2 218 1569

Email: [jamesmichael.b@chula.ac.th](mailto:jamesmichael.b@chula.ac.th)

Address: ChulaPhat-1 Building, 154 Rama 1 Road, Pathumwan, Bangkok, 10330, Thailand.

**Supplementary Figures**

**Supplementary Figure 1.** The effect of NBBA on cell viability of SHSY-5Y cells expressed as mean ± SEM. Statistical comparisons: 0.1% DMSO vs control or NBCA treatment (100, 200, 500, 600, 800, and 1000 nM); p value was obtained from one-way ANOVA followed by Dunnett’s *post hoc* multiple comparison test. **p < 0.01, ***p < 0.001. *n*=3.

**Supplementary Figure 2.** The effect of NCBA on cell viability of SHSY-5Y cells expressed as mean ± SEM. Statistical comparison: 0.1% DMSO vs control and NBBA treatments (100, 200, 500, 600, 800, and 1000nM) p value was obtained from one-way ANOVA followed by Dunnett’s *post hoc* multiple comparison test.: *p < 0.05, **p < 0.01, ***p < 0.001. *n*=3.

**Supplementary Figure 3.** Bright-field images corresponding to SH-SY5Y cell immunofluorescence images of NRF2 shown in main Figure 5, and supplementary Figure 4.

**Supplementary Figure 4.** NRF2-specific reactivity in immunofluorescence assay of SH-SY5Y cells in the presence and absence of H_2_O_2_ and/or NBCA.

**Supplementary Figure 5.** Bright field images corresponding to SH-SY5Y cell immunofluorescence images of S1R shown in main Figure 7 & Supplementary Figure 5.

**Supplementary Figure 6.** S1R specific reactivity in immunofluorescence assay of SHSY5Y Cells in the presence and absence of H_2_O_2_ and or NBCA.

**Supplementary Figure 7.** The specificity of S1R immunostaining was confirmed using primary antibody omission controls, which demonstrated negligible Alexa Fluor 488 fluorescence in the absence of the primary antibody

**Supplementary Figure 8.** Wild type N2A neurite outgrowth in presence of NBBA **A** wild type control **B** Wild type BD1047 5 µM **C** Wild type PRE084 5 µM. **D** NBBA 100 nM. **E** NBBA 100 µM + BD1047 5 µM **F** NBBA 300 nM **G** NBBA 300 nM + BD1047 5 µM **H** NBBA 500 nM. **I** NBBA 500 nM + BD1047 5 µM.

**Supplementary Figure 9.** S1R KO N2A neurite outgrowth in presence of NBBA **A** S1RKO N2A cells **B** S1RKO N2A BD1047 5 µM **C** S1RKO N2A PRE084 5 µM. **D** NBBA 100 nM. **E** NBBA 100 µM + BD1047 5 µM **F** NBBA 300 nM **G** NBBA 300 nM + BD1047 5 µM **H** NBBA 500 nM. **I** NBBA 500 nM + BD1047 5 µM

**Supplementary Figure 10.** Wild type N2A neurite outgrowth in presence of NBCA **A** wild type control **B** Wild type BD1047 5 µM **C** Wild type PRE084 5 µM. **D** NBCA 100 nM. **E** NBCA 100 µM + BD1047 5 µM **F** NBCA 300 nM **G** NBCA 300 nM + BD1047 5 µM **H** NBCA 500 nM. **I** NBCA 500 nM + BD1047 5 µM

**Supplementary Figure 11 S1R KO** N2A neurite outgrowth in presence of NBCA **A** S1RKO N2A cells **B** S1RKO N2A BD1047 5 µM **C** S1RKO N2A PRE084 5 µM. **D** NBCA 100 nM. **E** NBCA 100 µM + BD1047 5 µM **F** NBCA 300 nM **G** NBCA 300 nM + BD1047 5 µM **H** NBCA 500 nM. **I** NBCA 500 nM + BD1047 5 µM
